# Synthesis and Biological Evaluation of Phaeosphaeride A Derivatives as Antitumor Agents

**DOI:** 10.3390/molecules23113043

**Published:** 2018-11-21

**Authors:** Victoria Abzianidze, Petr Beltyukov, Sofya Zakharenkova, Natalia Moiseeva, Jennifer Mejia, Alvin Holder, Yuri Trishin, Alexander Berestetskiy, Victor Kuznetsov

**Affiliations:** 1 Laboratory of Chemical Modeling, Research Institute of Hygiene, Occupational Pathology and Human Ecology, Federal Medical Biological Agency, p/o Kuz’molovsky, 188663 Saint Petersburg, Russia; sofya.zakharenkova@gmail.com (S.Z.); kuznetsov_va1956@bk.ru (V.K.); 2 Laboratory of Molecular Toxicology and Experimental Therapy, Research Institute of Hygiene, Occupational Pathology and Human Ecology, Federal Medical Biological Agency, p/o Kuz’molovsky, 188663 Saint Petersburg, Russia; biochem2005@rambler.ru; 3 N.N. Blokchin National Medical Research Center of Oncology, 115478 Moscow, Russia; n.i.moiseeva@gmail.com; 4 Department of Chemistry and Biochemistry, Old Dominion University, 4541 Hampton Boulevard, Norfolk, VA 23529, USA; jmatt024@odu.edu (J.M.); aholder@odu.edu (A.H.); 5 Saint Petersburg State University of industrial technologies and design, Ivana Chernyh str., 4, 198095 Saint Petersburg, Russia; trish@yt4470.spb.edu; 6 All-Russian Institute of Plant Protection, Russian Academy of Agricultural Sciences, Pushkin, 196608 Saint Petersburg, Russia; aberestetski@yahoo.com

**Keywords:** natural phaeosphaeride A, antitumor activity, human tumor cell lines, HEF cell line, acute toxicity

## Abstract

New derivatives of phaeosphaeride A (PPA) were synthesized and characterized. Anti-tumor activity studies were carried out on the HCT-116, PC3, MCF-7, A549, К562, NCI-Н929, Jurkat, THP-1, RPMI8228 tumor cell lines, and on the HEF cell line. All of the compounds synthesized were found to have better efficacy than PPA towards the tumor cell lines mentioned. Compound **6** was potent against six cancer cell lines, HCT-116, PC-3, K562, NCI-H929, Jurkat, and RPMI8226, showing a 47, 13.5, 16, 4, 1.5, and 7-fold increase in anticancer activity comparative to those of etoposide, respectively. Compound **1** possessed selectivity toward the NCI-H929 cell line (IC_50_ = 1.35 ± 0.69 μM), while product **7** was selective against three cancer cell lines, HCT-116, MCF-7, and NCI-H929, each having IC_50_ values of 1.65 μM, 1.80 μM and 2.00 μM, respectively.

## 1. Introduction

Modern chemotherapeutic treatment of malignant tumors is widely considered to have begun approximately 75 years ago when researchers found that nitrogen mustard displayed anti-tumor activity [1]. In more recent decades, leading research approaches have focused on the development of new antitumor agents for targeted therapy, as well as combined treatment with immunotherapeutic and chemotherapeutic agents [2]. Diversity, and in many cases, the genetic uniqueness of tumors, does not allow for the development of universal and specific therapeutic treatment. As a result, the use of non-specific chemotherapeutic treatment is still the principal therapeutic option used in patients. Despite advances in cancer treatments, the disabling side effects and relative effectivity of these broad antitumor drugs plagues scientists to urgently develop improved chemotherapeutic agents.

Many chemotherapeutic agents used in clinical practice are developed from natural compounds or their derivatives and analogs, e.g., etoposide, eribulin, paclitaxel, vincristine, vinblastine, topotecan, cytarabine, doxorubicin, dactinomycin, and bleomycin [3,4,5]. These also include medicines developed and introduced into clinical practice from the 1950s–1960s (vincristine, vinblastine, cytarabine, etc.), as well as modern agents developed in the 2000’s (topotecan, eribulin). Many chemotherapeutic agents based on natural analogs continue to undergo clinical studies to evaluate their effectiveness in the treatment of various types of tumors, as drugs developed from natural compounds often show lower toxicity and have higher target values towards malignant tumor cells [3]. While scientists continue to research these compounds, these naturally derived drugs are already relevant in the market today, with close to 40% of antitumor drugs approved by the FDA being of natural origin or semi-synthetic derivatives of natural compounds. When we reexamine this number and consider only the use of synthetic drugs, the analogues of natural compounds, the proportion of these antitumor agents are estimated at 70% [6]. Clearly, nature remains an inexhaustible source of new substances and has vast potential to aid in the fight against various tumors.

Here, phaeosphaeride A, produced by endophytic fungi from the genus *Phaeosphaeria*, was chosen for its ability to inhibit the Signal Transducer and Activator of Transcription 3 (STAT3) signaling pathway. The stereochemical configuration of this natural product had been established by total synthesis of ent-phaeosphaeride A and phaeosphaeride A [7,8] and by X-ray diffraction [9]. High incidence of STAT3 protein is characteristic of several oncological diseases like leukemia, multiple myeloma, cancers of the breast and lung, as well as multiple carcinomas such as renal, prostate, hepatocellular, ovarian, and pancreatic [10,11]. STAT3 also is shown to play an important role in regulating cell growth and viability [10,12,13,14,15]. Therefore, phaeosphaeride A, and its derivatives, are potentially promising anticancer agents for targeted therapy and combined treatments [10,11].

The results of the study in this article include information on the methods for the synthesis of the phaeosphaeride A derivatives and their biological evaluation, including the measurements of the cytotoxic effects and a preliminary assessment of acute toxicity in mice.

## 2. Results and Discussion

### 2.1. Chemistry

**Scheme 1:**
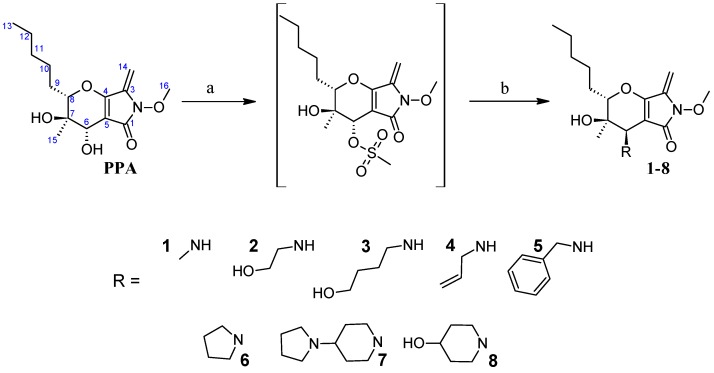
Synthesis of compounds **1**–**8**. *Reagents and conditions*: (**a**) MsCl, TEA, CH_2_Cl_2_, 0 °C, 1 h; (**b**) cyclic or primary amine, with or without TEA, acetonitrile or THF, room temperature or 70–75 °C.

The synthesis of target compounds **1**–**8** is presented in Scheme 1. Mesylation of PPA with MeSO_2_Cl and Et_3_N in CH_2_Cl_2_ gave the mesylate as a sole product, which was used in the next step without purification.

Treatment of the mesylate with cyclic amines and (or without) TEA in acetonitrile (or THF) at room temperature (or at 70–75 °C) or with primary amines in acetonitrile at room temperature gave the corresponding amino derivatives in 11–27% yield with inversion occurring on the C-6 atom. The ROESY spectra of the products showed a correlation between the methyl protons (H-15) and the H-6 proton confirming the inversion of configuration of the C-6 atom (see Supplementary Material). Product **6** was synthesized by our research group in 2017 with the present method doubling the total yield [16]. Previously it was impossible to obtain the desired derivatives through chloroacetyl PPA derivative from primary amines as those reactions proceeded solely at the exocyclic double bond (unpublished data) [17].

### 2.2. Biological Evaluation

#### 2.2.1. Cytotoxicity Assay Using 9 Tumor Cell Lines and HEF Cell Line

All newly synthesized PPA derivatives **1**–**8** were evaluated for their anti-proliferative activity against human breast cancer MCF-7, human prostate adenocarcinoma PC-3, human colorectal cancer HCT-116, human lung cancer A549, human chronic myelogenous leukemia K562, human acute monocytic leukemia THP-1, human multiple myeloma RPMI8226, human acute T-cell leukemia Jurkat, human multiple myeloma NCI-H929, and human embryonic fibroblasts HEF cell lines by MTT assays. All cells were incubated with different concentrations of PPA derivatives for 72 h, with etoposide and PPA used as reference compounds. The anticancer activity of the tested compounds was described as the concentration of drug inhibiting 50% cell growth IC_50_ (Table 1 and Table 2).

**Figure 1:**
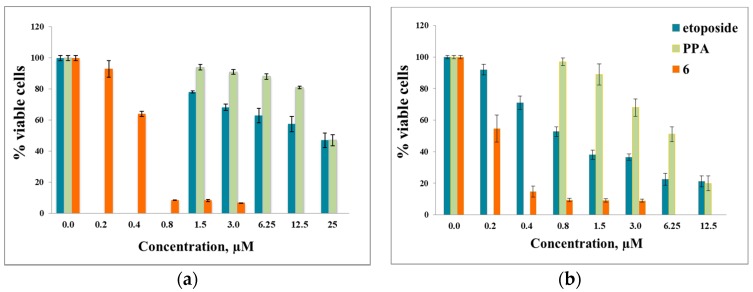
(**a**) The in vitro effects of compound **6** on the cell viability of human HCT-116 cell line; (**b**) The in vitro effects of compound **6** on the cell viability of human NCI-H929 cell line.

Compound **6** was found to be the most potent against six cancer cell lines (0.47 μM for HCT-116, 0.2 μM for PC-3, 0.54 μM for K562, 0.23 μM for NCI-H929, 0.55 μM for Jurkat and 0.63 μM for RPMI8226), which was 51, 160, 37, 28, 17.5, and 14.5-fold stronger than those of the reference compound PPA, respectively (Figure 1).

Compound **1** showed selectivity toward the NCI-H929 cell line (IC_50_ = 1.35 ± 0.69 μM) while product **7** was selective against three cancer cell lines (IC_50_ was 1.65 μM, 1.80 μM and 2.00 μM towards HCT-116, MCF-7 and NCI-H929, respectively). The results obtained indicate product **6** to be more toxic than the positive control, etoposide, against HCT-116 (47-fold), PC-3 (13.5-fold), K562 (16-fold), NCI-H929 (4-fold), Jurkat (1.5-fold) and RPMI8226 (7-fold) cancer cell lines. Human embryonic fibroblasts were used as a control.

#### 2.2.2. Acute Intraperitoneal Toxicity Study

No adverse effects were found from compound **6** on body weight and food consumption. There was no indication of morbidity, mortality, or lethal effects during the 14 days after i.p. administration (intraperiotenal) in mice of two low doses (8.25 and 82.5 mg/kg). LD50 was not reached in all experiments. Mortality (two of eight mice) was observed in the highest dose group (one was found deceased on Day 2 and one on Day 3). No effect was observed on mean body weight or mass coefficients (spleen, liver, heart, kidney, lung, thymus, and adrenal). No gross abnormalities were observed in the organs tested. Maximum tolerated dose of **6** after i.p. injection in mice was equal to or more than 82.5 mg/kg. Median lethal dose (LD50) of **6** has not been determined but is believed to be more than 200 mg/kg. It can be concluded that compound **6** is less toxic comparative to etoposide. Intraperitoneal LD50 of etoposide for mice is 64 mg/kg (RTECS # KC0190000).

## 3. Materials and Methods

### 3.1. Materials and Instruments

^1^H-NMR spectra were acquired on an AVANCE III 400 MHz NMR spectrometer (Bruker, Rheinstetten, Germany) in CDCl_3_. Optical rotations were acquired on a Polaar 3005 Polarimeter (Optical Activity, Huntingdon, Great Britain) using a 2.5 cm cell with a Na 589 nm filter and the concentration of samples was denoted as *c*. Mass spectra data were acquired on a TSQ Quantum Access Max Mass spectrometer (Thermo Fisher Scientific, Waltham, MA, USA). High-resolution mass spectra (HRMS) were acquired on a LTQ Orbitrap Velos spectrometer (Thermo Scientific) and on a Bruker MicrOTOF. FTIR spectra were acquired on an IR Affinity-1 spectrometer (Shimadzu, Thermo Scientific). Organic solvents used were dried by standard methods when necessary. Commercially available reagents were used without further purification. All reactions were monitored by TLC with silica gel coated plates (EMD/Merck KGaA, Darmstadt, Germany), with visualization by UV light and by charring with 0.1% ninhydrin in EtOH. Column chromatography was performed using Merck 60 Å 70–230 mesh silica gel. The optical density was determined using a Multiskan FC spectrophotometer (Thermo Scientific) at a wavelength of 540 nm when using the MTT assay.

### 3.2. Chemical Syntheses

#### 3.2.1. Synthesis of (2*S*,3*S*,4*S*)-3-Hydroxy-6-methoxy-3-methyl-7-methylene-5-oxo-2-pentyl-2,3,4,5,6,7-hexahydropyrano[2,3-*c*]pyrrol-4-yl methanesulfonate

PPA (1 mmol) was dissolved in CH_2_Cl_2_ (2 mL) and cooled to 0 °C. Triethylamine (3.5 mmol) was added followed by the dropwise addition of methanesulfonyl chloride (2.5 mmol). The mixture was stirred at 0 °C for 1 h. The reaction was quenched by the addition of saturated NaHCO_3_ solution and the mixture was extracted with dichloromethane (3 × 20 mL). The organic layer was washed with water and brine, dried over magnesium sulfate, and concentrated in vacuo after filtration. Mesylate was used in the next step without further purification. HRMS revealed an [M + H]^+^ion with exact mass 376.14219, corresponding to the molecular formula С_16_H_26_NO_7_S.

#### 3.2.2. General Procedure for the Synthesis of Compounds **1**–**5**

A mixture of the crude (2*S*,3*S*,4*S*)-3-hydroxy-6-methoxy-3-methyl-7-methylene-5-oxo-2-pentyl-2,3,4,5,6,7-hexahydropyrano[2,3-*c*]pyrrol-4-yl methanesulfonate (1 mmol) and an appropriate primary amine (2 mmol) was stirred in dry acetonitrile (2 mL) at room temperature until consumption of the starting material was complete as judged by TLC analysis (24–48 h). The reaction mixture was diluted with ether (20 mL) and transferred to a separatory funnel. The layers were separated and the aqueous layer was extracted with ether (3 × 20 mL). The organic extracts were combined, washed with brine (2 × 20 mL), dried over magnesium sulfate, and concentrated in vacuo. The crude product was purified by flash chromatography (DCM:methanol = 60:1).

*(2S,3R,4R)-3-Hydroxy-6-methoxy-3-methyl-4-(methylamino)-7-methylene-2-pentyl-3,4,6,7-tetrahydro-pyrano[2,3-c]pyrrol-5(2H)-one* (**1**): Yield 17%, yellow oil. ${\left[\alpha \right]}_{D}^{20.0}$ = −120.36 (c 0.28, CH_2_Cl_2_). ^1^H-NMR (CDCl_3_) δ 5.56 (m, 2H), 5.12 (s, 1H), 5.07 (s, 1H), 4.05 (m, 1H), 3.93 (s, 3H), 3.41 (m, 1H), 2.75 (s, 3H), 1.93 (m, 1H), 1.63 (m, 2H), 1.43–1.33 (m, 5H), 1.15 (s, 3H), 0.90 (m, 3H). ^13^C-NMR (CDCl_3_) δ 165.87 (s), 159.47 (s), 136.05 (s), 99.07 (s), 93.27 (s), 81.54 (s), 68.97 (s), 64.70 (s), 57.32 (s), 33.50 (s), 31.58 (s), 27.62 (s), 25.99 (s), 22.53 (s), 19.45 (s), 14.05 (s). IR (KBr) 3319, 2955, 2930, 2859, 1721, 1667, 1633, 1438, 1379, 1266, 1196, 1166, 1085, 979, 915 cm^−1^. HRMS [M + H]^+^ calcd for C_16_H_27_N_2_O_4_ 311.19653, found 311.1953.

*(2S,3R,4R)-3-Hydroxy-4-[(2-hydroxyethyl)amino]-6-methoxy-3-methyl-7-methylene-2-pentyl-3,4,6,7-tetra-hydropyrano[2,3-c]pyrrol-5(2H)-one* (**2**): Yield 25%, yellow oil. ${\left[\alpha \right]}_{D}^{20.0}$ = −99.54 (c 0.22, CH_2_Cl_2_). ^1^H-NMR (CDCl_3_) δ 5.09 (s, 1H), 5.04 (s, 1H), 3.93 (m, 4H), 3.61 (m, 2H), 3.12 (s, 1H), 3.06–2.91 (m, 2H), 1.94 (m, 1H), 1.65–1.58 (m, 2H), 1.35 (m, 6H), 1.06 (s, 3H), 0.91 (s, 3H). ^13^C-NMR (CDCl_3_) δ 167.38 (s), 157.36 (s), 136.27 (s), 104.10 (s), 92.86 (s), 82.53 (s), 69.28 (s), 64.71 (s), 61.07 (s), 54.14 (s), 52.07 (s), 31.66 (s), 27.47 (s), 26.28 (s), 22.57 (s), 18.58 (s), 14.08 (s). IR (KBr) 3322, 2954, 2927, 2857, 1718, 1633, 1547, 1458, 1437, 1378, 1263, 1191, 1117, 1081, 978, 914 cm^−1^. HRMS [M + H]^+^ calcd for C_17_H_29_N_2_O_5_ 341.20710, found 341.2060.

*(2S,3R,4R)-3-Hydroxy-4-[(4-hydroxybutyl)amino]-6-methoxy-3-methyl-7-methylene-2-pentyl-3,4,6,7-tetra-hydropyrano[2,3-c]pyrrol-5(2H)-one* (**3**): Yield 20%, yellow oil. ${\left[\alpha \right]}_{D}^{20.0}$ = −89.80 (c 0.51, CH_2_Cl_2_). ^1^H-NMR (CDCl_3_) δ 5.11 (d, *J* = 1.2 Hz, 1H), 5.05 (d, *J* = 1.3 Hz, 1H), 4.94 (m, 3H), 4.05 (m, 1H), 3.93 (s, 3H), 3.80–3.50 (m, 2H), 3.36 (s, 1H), 3.27–3.12 (m, 1H), 3.06–2.87 (m, 1H), 1.95 (m, 1H), 1.78 (m, 2H), 1.69–1.55 (m, 4H), 1.45–1.33 (m, 5H), 1.12 (s, 3H), 0.90 (m, 3H). ^13^C-NMR (CDCl_3_) δ 166.37 (s), 158.18 (s), 136.38 (s), 102.16 (s), 92.59 (s), 82.14 (s), 68.78 (s), 64.64 (s), 62.26 (s), 55.97 (s), 48.77 (s), 31.64 (s), 30.14 (s), 27.61 (s), 26.12 (s), 22.57 (s), 19.15 (s), 14.08 (s). IR (KBr) 3292, 2927, 2858, 1712, 1632, 1548, 1438, 1379, 1191, 1083, 989, 914 cm^−1^. HRMS [M + H]^+^ calcd for C_19_H_33_N_2_O_5_ 369.23840, found 369.2373.

*(2S,3R,4R)-4-(Allylamino)-3-hydroxy-6-methoxy-3-methyl-7-methylene-2-pentyl-3,4,6,7-tetrahydropyrano-[2,3-c]pyrrol-5(2H)-one* (**4**): Yield 14%, yellow oil. ${\left[\alpha \right]}_{D}^{20.0}$ = −157.50 (c 0.31, CH_2_Cl_2_). ^1^H-NMR (CDCl_3_) δ 5.89 (ddt, *J* = 12.2, 10.1, 6.1 Hz, 1H), 5.25 (dd, *J* = 17.2, 1.3 Hz, 1H), 5.12 (m, 1H), 5.03 (d, *J* = 1.3 Hz, 1H), 4.99 (d, *J* = 1.3 Hz, 1H), 3.93 (s, 3H), 3.78–3.66 (m, 1H), 3.58–3.49 (m, 1H), 3.44 (ddd, *J* = 13.8, 4.6, 1.2 Hz, 1H), 3.03 (s, 1H), 1.94 (m, 1H), 1.60 (m, 2H), 1.43–1.35 (m, 5H), 1.06 (s, 3H), 0.91 (m, 3H). ^13^C-NMR (CDCl_3_) δ 166.68 (s), 156.86 (s), 136.78 (s), 136.06 (s), 117.05 (s), 105.17 (s), 91.79 (s), 82.65 (s), 68.23 (s), 64.59 (s), 54.92 (s), 52.14 (s), 31.69 (s), 27.63 (s), 26.29 (s), 22.57 (s), 18.72 (s), 14.09 (s). IR (KBr) 3314, 2955, 2928, 2858, 1720, 1668, 1634, 1437, 1367, 1232, 1192, 1087, 992, 919 cm^−1^. HRMS [M + H]^+^ calcd for C_18_H_29_N_2_O_4_ 337.21218, found 337.2111.

*(2S,3R,4R)-4-(benzylamino)-3-hydroxy-6-methoxy-3-methyl-7-methylene-2-pentyl-3,4,6,7-tetrahydro-pyrano[2,3-c]pyrrol-5(2H)-one* (**5**): Yield 17%, yellow oil. ${\left[\alpha \right]}_{D}^{20.0}$ = −137.81 (c 0.43, CH_2_Cl_2_). ^1^H-NMR (CDCl_3_) δ 7.39–7.26 (m, 5H), 5.04 (d, *J* = 1.4 Hz, 1H), 5.00 (d, *J* = 1.4 Hz, 1H), 4.30 (d, *J* = 12.3 Hz, 1H), 3.94 (m, 4H), 3.53 (m, 1H), 3.12 (s, 1H), 1.93 (m, 1H), 1.61 (m, 2H), 1.34 (m, 5H), 1.06 (s, 3H), 0.90 (m, 3H). ^13^C-NMR (CDCl_3_) δ 166.78 (s), 156.89 (s), 139.35 (s), 136.81 (s), 128.61 (d, *J* = 8.7 Hz), 127.47 (s), 105.30 (s), 91.89 (s), 82.70 (s), 68.26 (s), 64.63 (s), 55.37 (s), 53.62 (s), 31.68 (s), 27.63 (s), 26.27 (s), 22.56 (s), 18.82 (s), 14.07 (s). IR (KBr) 3309, 2955, 2927, 2858, 1720, 1633, 1454, 1436, 1378, 1232, 1192, 1086, 978, 914 cm^−1^. HRMS [M + H]^+^ calcd for C_22_H_31_N_2_O_4_ 387.22783, found 387.2262.

#### 3.2.3. General Procedure for the Synthesis of Compounds **6**–**8**

A mixture of the crude (2*S*,3*S*,4*S*)-3-hydroxy-6-methoxy-3-methyl-7-methylene-5-oxo-2-pentyl-2,3,4,5,6,7-hexahydropyrano[2,3-*c*]pyrrol-4-yl methanesulfonate (1 mmol), the corresponding cyclic amine (1.5 mmol) and triethylamine (3 mmol) was stirred in dry acetonitrile (2 mL) at room temperature until consumption of the starting material was complete as judged by TLC analysis (24-48 h). The reaction mixture was quenched with water and extracted with EtOAc (2 × 20 mL). The organic extract was washed with brine, dried over magnesium sulfate, and concentrated in vacuo. The crude product was purified by flash chromatography (DCM/methanol).

#### 3.2.4. Alternative General Procedure for the Synthesis of Compounds **6**–**8**

A mixture of the crude (2*S*,3*S*,4*S*)-3-hydroxy-6-methoxy-3-methyl-7-methylene-5-oxo-2-pentyl-2,3,4,5,6,7-hexahydropyrano[2,3-*c*]pyrrol-4-yl methanesulfonate (1 mmol) and the corresponding cyclic amine (5 mmol) in dry THF (2 mL) was heated at 70–75 °C in a sealed tube for 18 h. The mixture was cooled to room temperature, filtered, and the THF solution was diluted with saturated sodium bicarbonate solution (20 mL). The resulting mixture was extracted with DCM (3 × 20 mL). The organic extracts were washed with water, dried with magnesium sulfate, and concentrated in vacuo. The crude product was purified by flash chromatography (DCM/methanol).

*(2S,3R,4R)-3-Hydroxy-6-methoxy-3-methyl-7-methylene-2-pentyl-4-pyrrolidin-1-yl-3,4,6,7-tetrahydro-pyrano[2,3-c]pyrrol-5(2H)-one* (**6**): Yield 27%, yellow oil. ${\left[\alpha \right]}_{D}^{20.0}$ = −176.52 (c 0.40, CH_2_Cl_2_). ^1^H-NMR (CDCl_3_) δ 5.40 (s, 1H), 5.02 (d, *J* = 1.4 Hz, 1H), 4.98 (d, *J* = 1.4 Hz, 1H), 3.92 (s, 3H), 3.64 (d, *J* = 9.9 Hz, 1H), 3.31 (s, 1H), 3.10–2.55 (m, 4H), 2.03–1.89 (m, 1H), 1.84–1.70 (m, 4H), 1.69–1.52 (m, 2H), 1.45–1.29 (m, 5H), 1.05 (s, 3H), 0.91 (t, *J* = 6.7 Hz, 3H). ^13^C-NMR (CDCl_3_) δ 167.11 (s), 158.44 (s), 137.00 (s), 101.65 (s), 91.38 (s), 83.60 (s), 68.00 (s), 64.41 (s), 59.55 (s), 31.73 (s), 28.21 (s), 26.43 (s), 23.91 (s), 22.55 (s), 19.66 (s), 14.04 (s). IR (KBr) 3430, 2957, 2930, 2860, 1722, 1633, 1438, 1190, 1144 cm^−1^. HRMS [M + H]^+^, calcd. for C_19_H_31_N_2_O_4_ 351.227834, found 351.22733.

*(2S,3R,4R)-3-Hydroxy-6-methoxy-3-methyl-7-methylene-2-pentyl-4-(4-pyrrolidin-1-ylpiperidin-1-yl)-3,4,6,7-tetrahydropyrano[2,3-c]pyrrol-5(2H)-one* (**7**): Yield 11%, yellow oil. ${\left[\alpha \right]}_{D}^{20.0}$ = −115.82 (c 0.41, CH_2_Cl_2_). ^1^H-NMR (CDCl_3_) δ 5.05 (s, 1H), 5.00 (s, 1H), 3.91 (s, 3H), 3.56 (m, 1H), 3.29–2.91 (m, 8H), 2.81 (m, 1H), 2.65 (s, 1H), 2.27 (t, *J* = 10.9 Hz, 1H), 2.11–1.83 (m, 9H), 1.69–1.48 (m, 2H), 1.35 (s, 5H), 1.02 (s, 3H), 0.91 (s, 3H). ^13^C-NMR (CDCl_3_) δ 167.08 (s), 158.19 (s), 136.87 (s), 101.35 (s), 91.69 (s), 83.39 (s), 68.22 (s), 64.49 (s), 62.51 (s), 61.35 (s), 55.67 (s), 51.39 (s), 49.52 (s), 32.19 (s), 31.74 (s), 28.29 (s), 26.44 (s), 23.20 (s), 22.57 (s), 19.96 (s), 14.08 (s). IR (KBr) 3213, 2928, 2857, 2782, 1724, 1669, 1635, 1437, 1377, 1354, 1321, 1193, 1150, 1126, 1085, 979 cm^−1^. HRMS [M + H]^+^ calcd for C_24_H_40_N_3_O_4_ 434.30133, found 434.3009.

*(2S,3R,4R)-3-Hydroxy-4-(4-hydroxypiperidin-1-yl)-6-methoxy-3-methyl-7-methylene-2-pentyl-3,4,6,7-tetra-hydropyrano[2,3-c]pyrrol-5(2H)-one* (**8**): Yield 16%, yellow oil. ${\left[\alpha \right]}_{D}^{20.0}$ = −111.11 (c 0.36, CH_2_Cl_2_). ^1^H-NMR (CDCl_3_) δ 5.17 (s, 1H), 5.03 (d, *J* = 1.1 Hz, 1H), 5.00 (d, *J* = 1.1 Hz, 1H), 3.93 (s, 3H), 3.68 (s, 1H), 3.59 (m, 1H), 3.02 (s, 1H), 2.87 (s, 2H), 2.35 (s, 1H), 2.03–1.85 (m, 3H), 1.68–1.50 (m, 6H), 1.35 (m, 5H), 1.05 (s, 3H), 0.91 (m, 3H). ^13^C-NMR (CDCl_3_) δ 167.01 (s), 158.15 (s), 136.85 (s), 101.35 (s), 91.75 (s), 83.35 (s), 68.20 (s), 64.53 (s), 62.45 (s), 35.20 (s), 31.79 (s), 28.35 (s), 26.48 (s), 22.58 (s), 20.00 (s), 14.09 (s). IR (KBr) 3213, 2926, 2855, 1720, 1671, 1634, 1547, 1438, 1375, 1139, 1066, 1045 cm^−1^. HRMS [M + H]^+^ calcd for C_20_H_33_N_2_O_5_ 381.23840, found 381.2377.

### 3.3. Bio-Evaluation Methods

#### 3.3.1. In-Vitro Cytotoxicity Study (MTT Assay)

MCF-7 (breast cancer), PC-3 (prostate adenocarcinoma), HCT-116 (colorectal cancer cell), A549 (lung cancer), K562 (chronic myelogenous leukemia), THP-1 (acute monocytic leukemia), and RPMI8226 (multiple myeloma), Jurkat (acute T-cell leukemia), HEF (human embryonic fibroblasts) was purchased from the Russian Academy of Sciences Cells Bank (Institute of Cytology of the Russian Academy of Sciences, Saint Petersburg, Russian Federation). Multiple myeloma cell line NCI-H929 was purchased from ATCC (USA). The MCF-7, A549 and HEF (human embryonic fibroblasts) were cultured in DMEM medium. Other cell lines were cultured in RPMI1640 medium, (PanEco, Russia) supplemented with 10% fetal bovine serum (GE Healthcare LifeSciences, São Paulo, Brazil), and gentamicin at a concentration of 40 μg/mL and cultured at 37 °C in a humidified atmosphere containing 5% CO_2_. All experiments were performed with cells at passages 3 to 7 in the logarithmic phase of growth.

Cells were seeded into 96-well plates of 5 × 10^3^ for adhesive cultures and 20 × 10^3^ for suspension cultures per well in 90 μL and 135 μL of culture medium, respectively. The test substances were dissolved in DMSO to a concentration of 1 × 10^−2^ M. For subsequent dilution of the substances, a serum-free culture medium was used as the diluent. Final concentration of DMSO in wells was no more than 1%. It was found that this concentration of DMSO didn’t affect the cells. Substances were added to the cells in 3–4 replicates after 24 h for adhesive cultures and immediately for suspension cultures. 10–15 μL of serum-free medium was added to the control wells with non-exposed cells. Cells were cultured for 72 h at 37 °C in an atmosphere of 5% CO_2_.

#### 3.3.2. Experimental Animals

Six to eight-week-old outbreed male mice were purchased from the Rappolovo Animal Farm of the Russian Academy of Sciences. Animals were group housed in solid bottom polycarbonate cages (3–5 animals/cage) and provided with sterilized pelleted food and pure water ad libitum.

#### 3.3.3. Ethical Guidelines

Animal care and protocols of the study were conducted in compliance with ethical standards and recommendations for the human treatment of animals used in experimental and other scientific purposes according to the European Convention for the Protection of Vertebrate Animals used for Experimental and other Scientific Purposes (and protocol of amendment ETS No 170), the National Standard of the Russian Federation GOST R-53434-2009, "Principles of Good Laboratory Practice," and by the order of the Ministry of Health of the Russian Federation 01.04.2016, No 199n «On approval of the rules of good laboratory practice».

#### 3.3.4. Acute Intraperitoneal Toxicity Study

Thirty mice were randomly assigned to the acute toxicity study. Animals in experimental groups (8 per/group) received a dose formulation containing compound **6** solution in 5% DMSO at various dosages (8.25, 82.5 and 200 mg/kg) via single i.p. injection. Mice in the control group treated with vehicle (5% DMSO). The location of the i.p. injection was in the lower left abdominal quadrant. Volume of injection did not exceed 250 µL. After treatments, the following parameters and end points were evaluated for 14 days: mortality, clinical signs, body weight, food consumption, locomotion, salivation, diarrhea, and lethargy. The maximum tolerated dose in this study is defined as the highest dose that will be tolerated and not produce major life-threatening toxicity for the study duration [18]. All experimental procedures were performed according to the principles and guidelines for the care and use of laboratory animals. The animals were sacrificed by carbon dioxide asphyxiation and cervical dislocation. All efforts were made to minimize suffering. Post mortem evaluation included gross examination for all animals at terminal necropsy and calculation of organ mass coefficients. No histopathological examinations were performed.

## 4. Conclusions

In summary, a series of PPA derivatives were synthesized via mesylation and amination. Some of these derivatives represent a promising class of cytotoxic agents with potential therapeutic values. Compound **6** demonstrated the highest cytotoxicity and was less toxic when used in vivo compared with the clinically used etoposide.

## Figures and Tables

**Table 1:** IC_50_ values for the respective compounds when studied on the adhesive cell lines. Data was expressed as the inhibitory ratio ± SD based on three independent experiments (*n* = 3).

Compound	Adhesive Cell Cultures, IC_50_ (μM)				
	HCT-116	PC-3	MCF-7	A549	HEF
**PPA**	24.21 ± 0.75	32.14 ± 0.77	20.30 ± 0.8	41.10 ± 2.6	19.05 ± 0.25
**1**	3.68 ± 0.81	3.35 ± 0.92	4.10 ± 0.44	12.73 ± 0.40	22.30 ± 0.44
**2**	4.63 ± 0.04	5.55 ± 1.48	3.23 ± 1.07	15.14 ± 0.45	16.20 ± 0.26
**3**	2.90 ± 0.98	4.50 ± 1.56	3.05 ± 1.20	11.41 ± 0,19	15.19 ± 1.02
**4**	8.40 ± 0.24	6.37 ± 0.18	4.13 ± 0.23	12.65 ± 0.27	5.03 ± 0.15
**5**	12.93 ± 0.30	24.67 ± 1.24	35.71 ± 0.65	37.51 ± 1.33	53.11 ± 1.06
**6**	0.47 ± 0.01	0.20 ± 0.07	3.25 ± 0.64	10.11 ± 0.5	4.00 ± 0.17
**7**	1.65 ± 0.63	3.65 ± 0.64	1.80 ± 0.44	12.22 ± 0.2	22.30 ± 0.33
**8**	2.64 ± 0.05	4.53 ± 0.40	3.20 ± 0.52	9.40 ± 0.14	6.70 ± 0.23
**Etoposide**	22.00 ± 1.10	2.70 ± 0.05	9.60 ± 0.27	>100	>100

**Table 2:** IC_50_ values for the respective compounds when studied on the suspension cell lines. Data were expressed as inhibitory ratio ± SD based on three independent experiments (*n* = 3).

Compound	Suspension Cell Cultures, IC_50_ (μM)				
	К562	NCI-Н929	Jurkat	THP-1	RPMI8228
**PPA**	20.47 ± 1.46	6.50 ± 0.30	9.70 ± 0.42	19.10 ± 0.45	9.15 ± 0.64
**1**	3.25 ± 0.64	**1.35 ± 0.69**	2.75 ± 0.21	2.25 ± 0.21	3.97 ± 0.68
**2**	5.50 ± 0.57	2.05 ± 0.35	2.60 ± 0.99	2.30 ± 0.57	3.50 ± 0.82
**3**	6.70 ± 0.28	1.95 ± 0.21	3.15 ± 1.77	2.60 ± 0.57	2.70 ± 0.28
**4**	10.48 ± 0.41	2.35 ± 0.09	3.27 ± 0.07	3.32 ± 0.10	6.00 ± 0.13
**5**	14.13 ± 0.48	7.73 ± 0,25	10.10 ± 0.44	15.11 ± 0.36	16 ± 0.16
**6**	**0.54 ± 0.03**	**0.23 ± 0.02**	**0.55 ± 0.29**	2.05 ± 0.21	**0.63 ± 0.23**
**7**	6.03 ± 0.91	**2.00 ± 0.26**	2.73 ± 1.53	3.45 ± 0.21	3.35 ± 1.48
**8**	4.90 ± 2.40	1.87 ± 0.25	2.60 ± 1.13	2.10 ± 0.28	1.40 ± 0.28
**Etoposide**	8.47 ± 0.95	0.92 ± 0.03	0.88 ± 0.74	0.83 ± 0.21	4.60 ± 0.28

## Supplementary Materials

^1^H-NMR, ^13^C-NMR, ^1^H-^1^H ROESY and HRMS of these compounds are available in the supplementary materials.
