# Supplementary material for: Synthesis and Biological Evaluation of Phaeosphaeride A Derivatives as Antitumor Agents
Source: Molecules. 2018 Nov 21;23(11):3043. doi: 10.3390/molecules23113043 (PMC6278656; doi:10.3390/molecules23113043)
Supplement: Supplementary file 1 [file molecules-23-03043-s001.pdf]

Synthesis and Biological Evaluation of Phaeosphaeride A Derivatives as Antitumor Agents

Victoria Abzianidze, Petr Beltyukov, Sofya Zakharenkova, Natalia Moiseeva, Jennifer Mejia, Alvin Holder, Yuri Trishin, Alexander Berestetskiy and Victor Kuznetsov

Copy of ^{1}H-NMR, ^{13}C-NMR, ^{1}H-^{1}H ROESY and HRMS……………………………………………………2-19

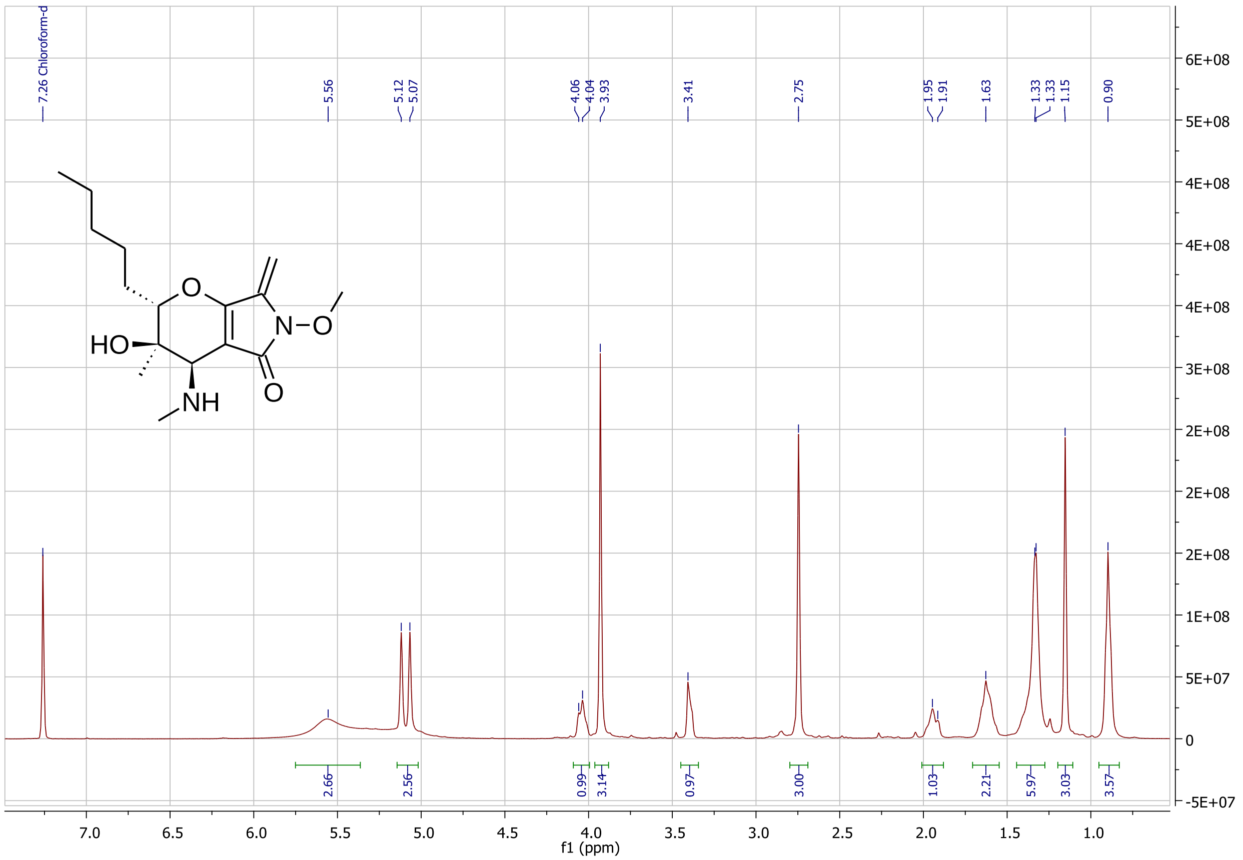

Figure 1. ^{1}H-NMR of **1**.

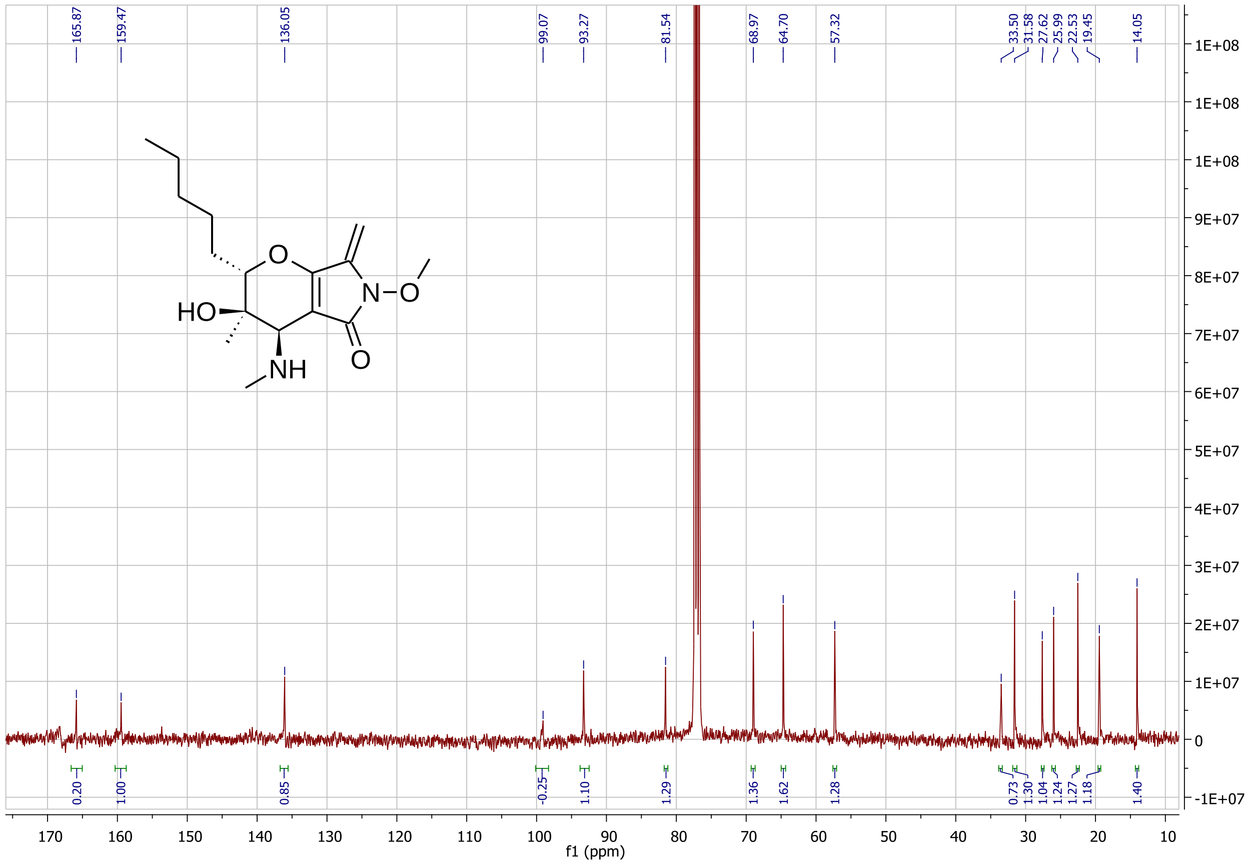

Figure 2. ^{13}C-NMR of **1**.

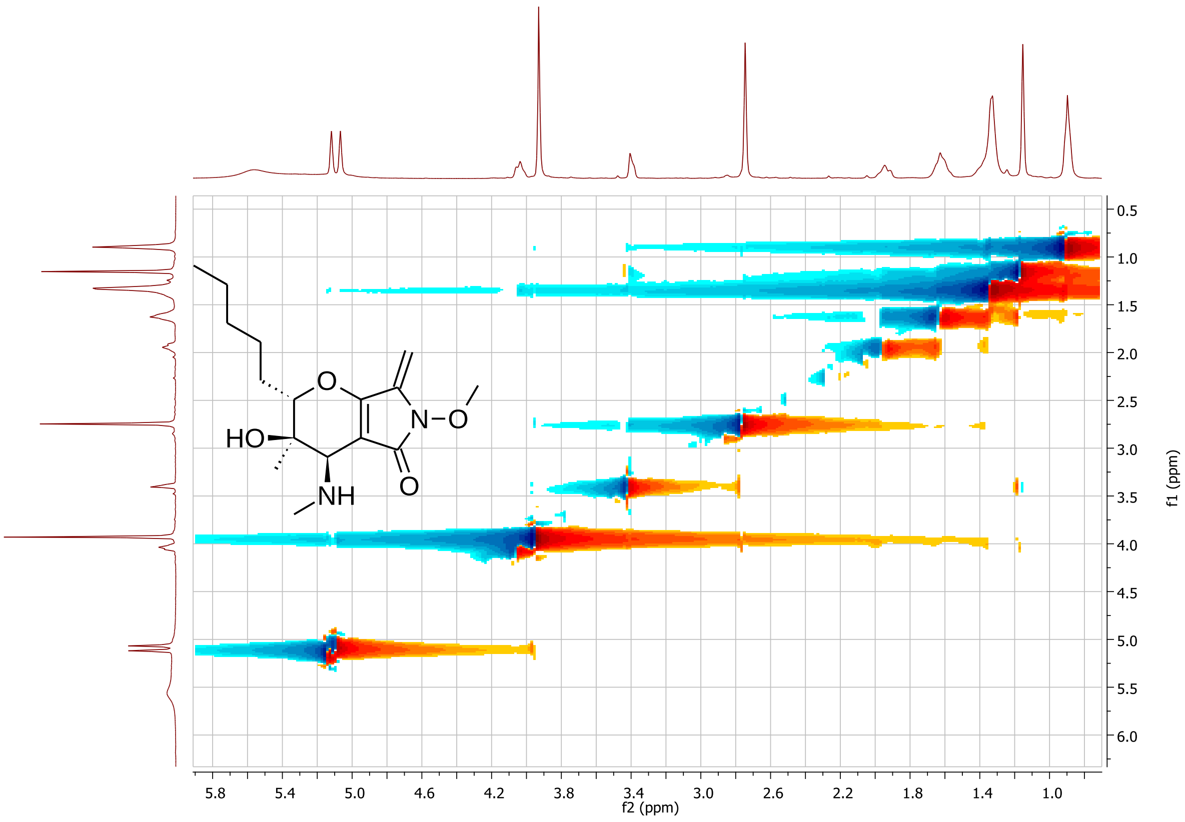

Figure 3. [1]H-^{1}H ROESY of **1**.

Display Report

Analysis Info

Analysis Name	D:\Data\2018\february\08\abz15.d	Acquisition Date	08.02.2018 18:59:24
Method	tune_low.m	Operator	Bruker Customer
Sample Name	abz15	Instrument / Ser#	micrOTOF 10223
Comment	MEOH 50v		

Acquisition Parameter

Source Type	ESI	Ion Polarity	Positive	Set Nebulizer	0.4 Bar
Focus	Not active			Set Dry Heater	180 °C
Scan Begin	50 m/z	Set Capillary	4500 V	Set Dry Gas	4.0 l/min
Scan End	3000 m/z	Set End Plate Offset	-500 V	Set Divert Valve	Source

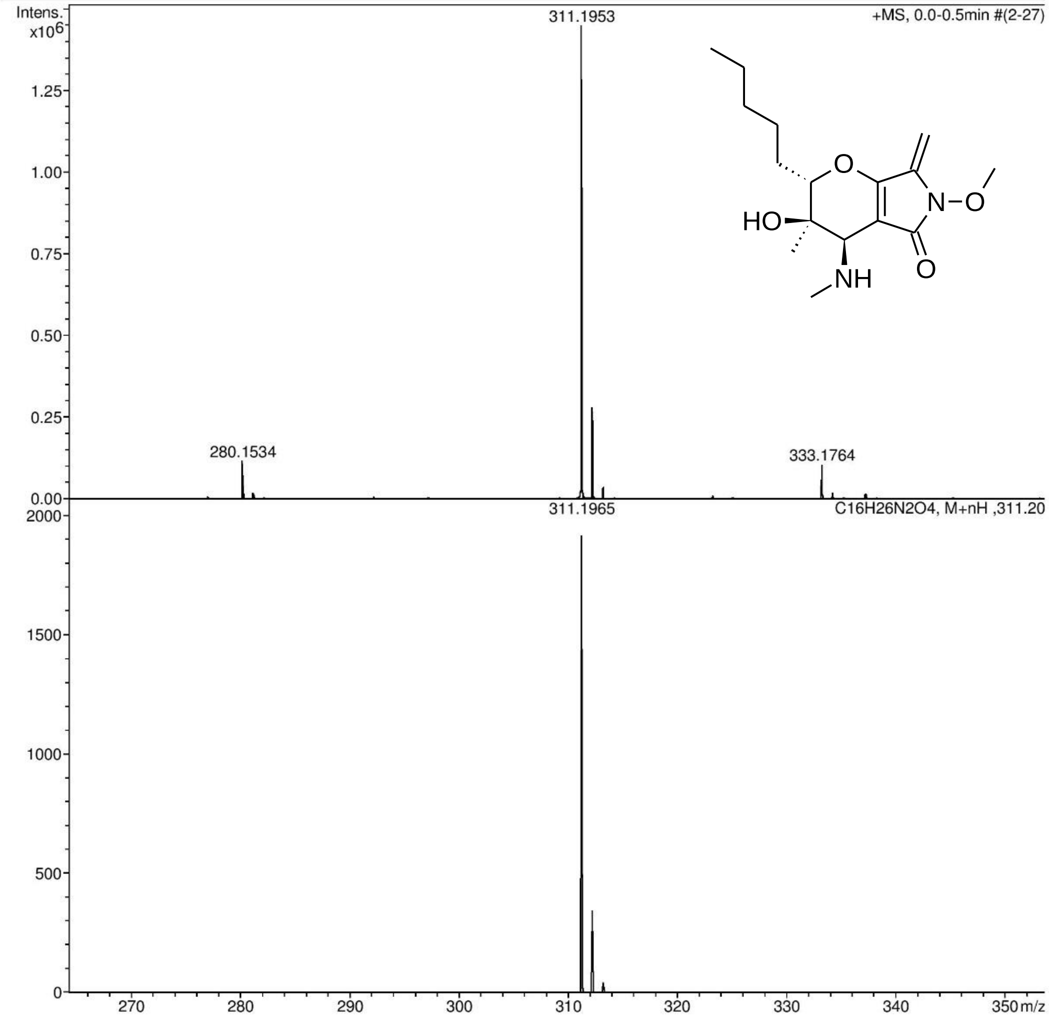

Bruker Compass DataAnalysis 4.0 printed: 08.02.2018 19:02:30 Page 1 of 1

Figure 4. HRMS of **1**.

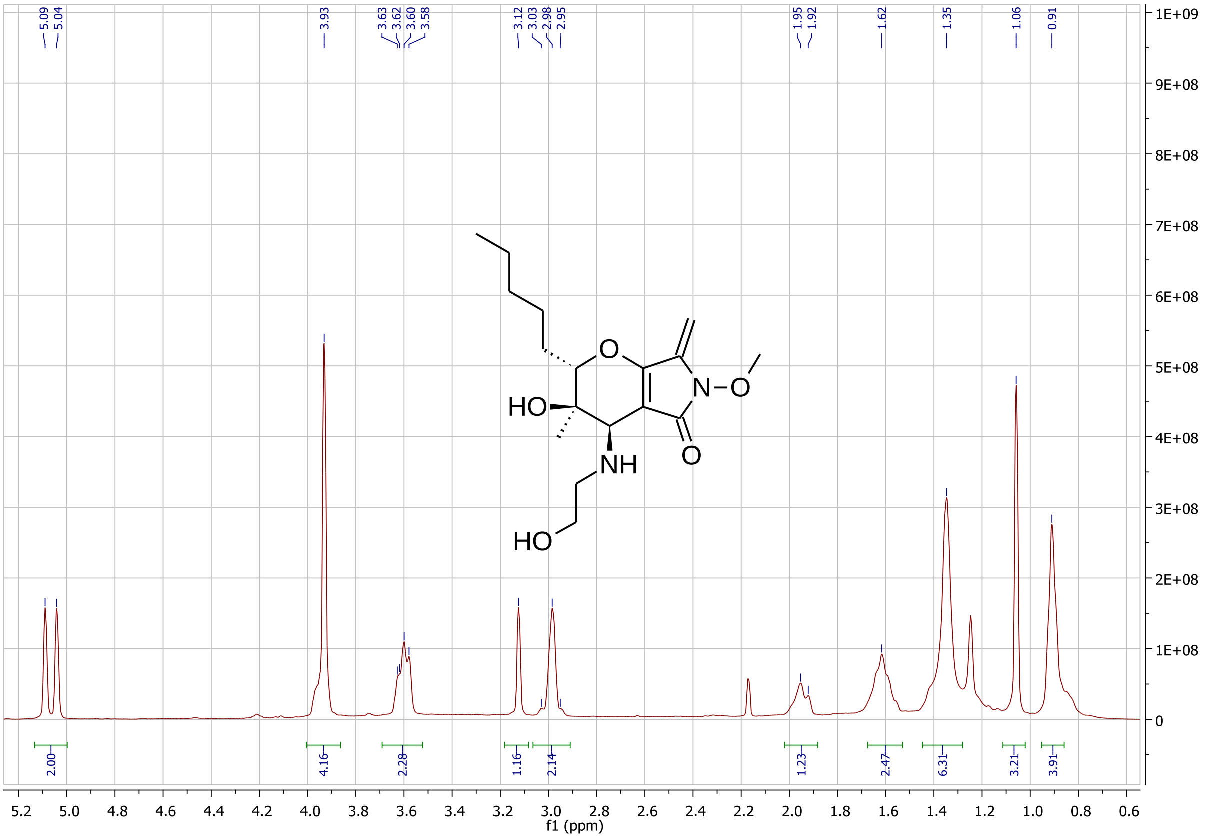

Figure 5. ^{1}H-NMR of **2**.

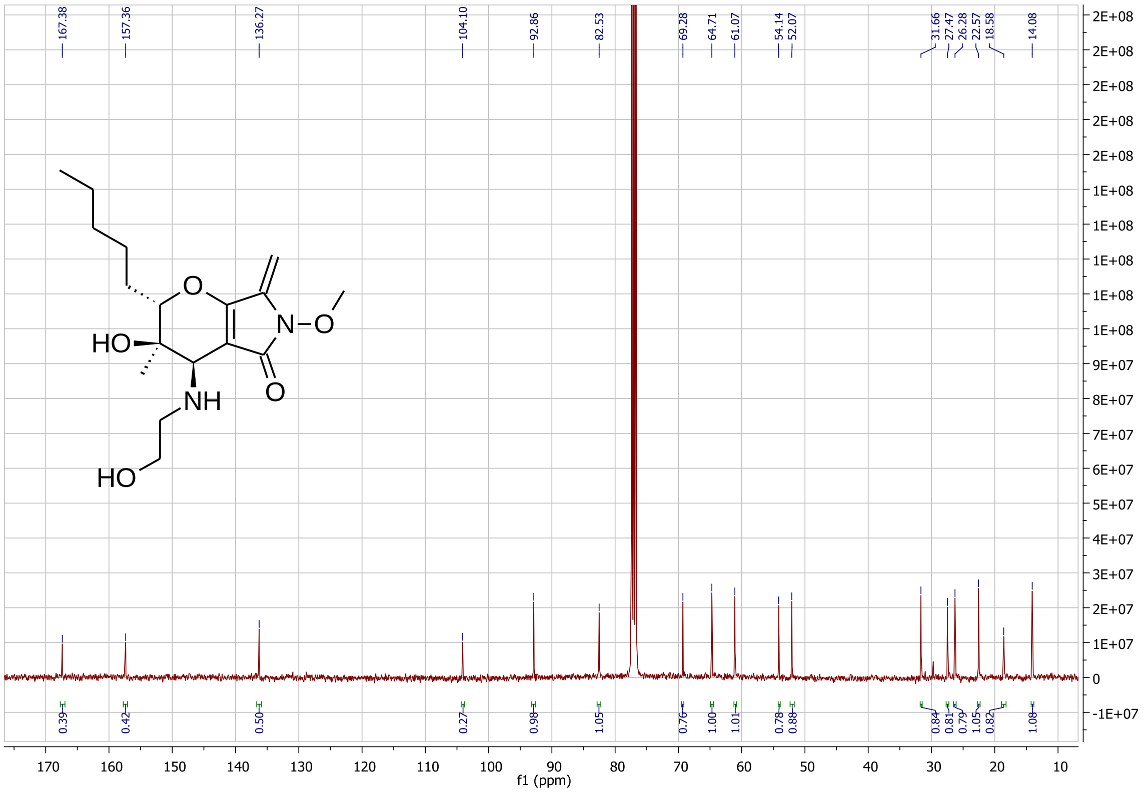

Figure 6. ^{13}C-NMR of **2**.

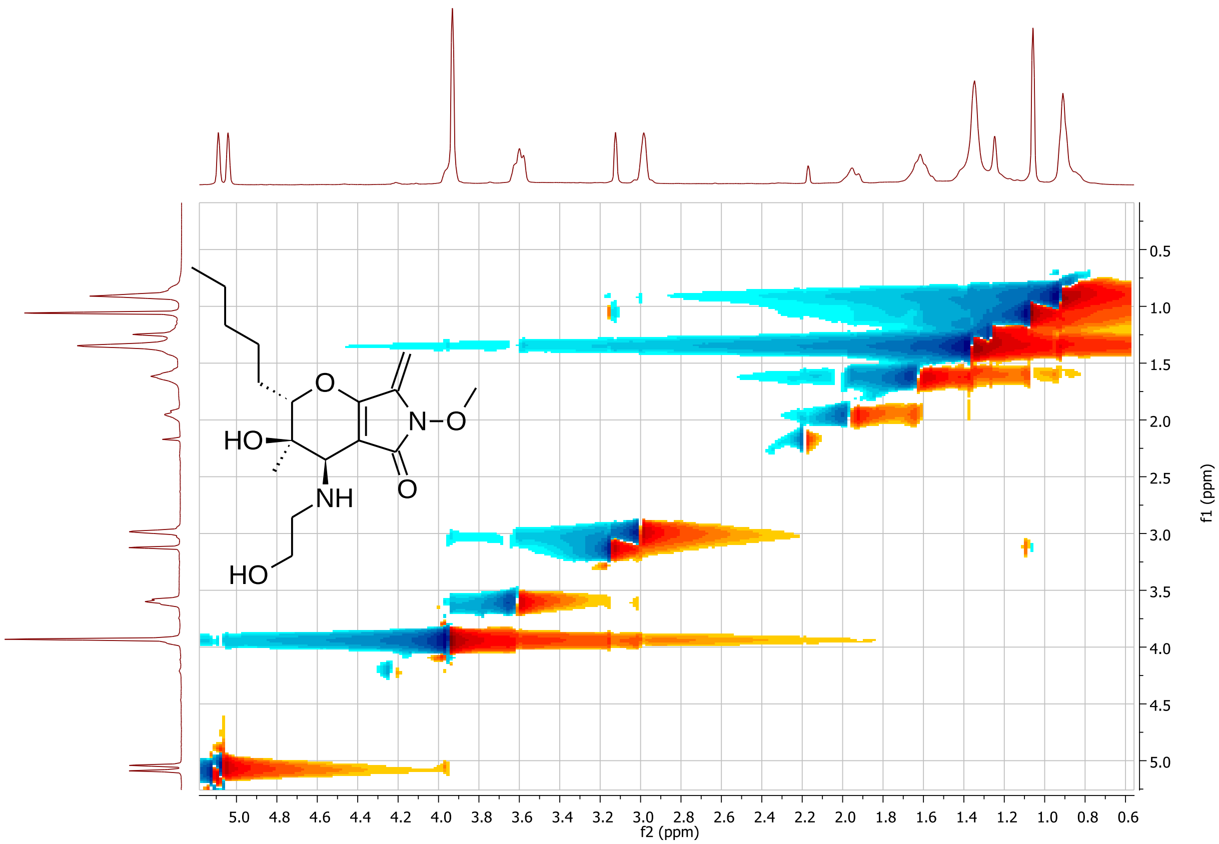

Figure 7. ^{1}H-^{1}H ROESY of **2**.

Display Report

Analysis Info

Analysis Name	D:\Data\2018\february\08\abz16.d	Acquisition Date	08.02.2018 19:15:13
Method	tune_low.m	Operator	Bruker Customer
Sample Name	abz16	Instrument / Ser#	micrOTOF 10223
Comment	MEOH		

Acquisition Parameter

Source Type	ESI	Ion Polarity	Positive	Set Nebulizer	0.4 Bar
Focus	Not active			Set Dry Heater	180 °C
Scan Begin	50 m/z	Set Capillary	4500 V	Set Dry Gas	4.0 l/min
Scan End	3000 m/z	Set End Plate Offset	-500 V	Set Divert Valve	Source

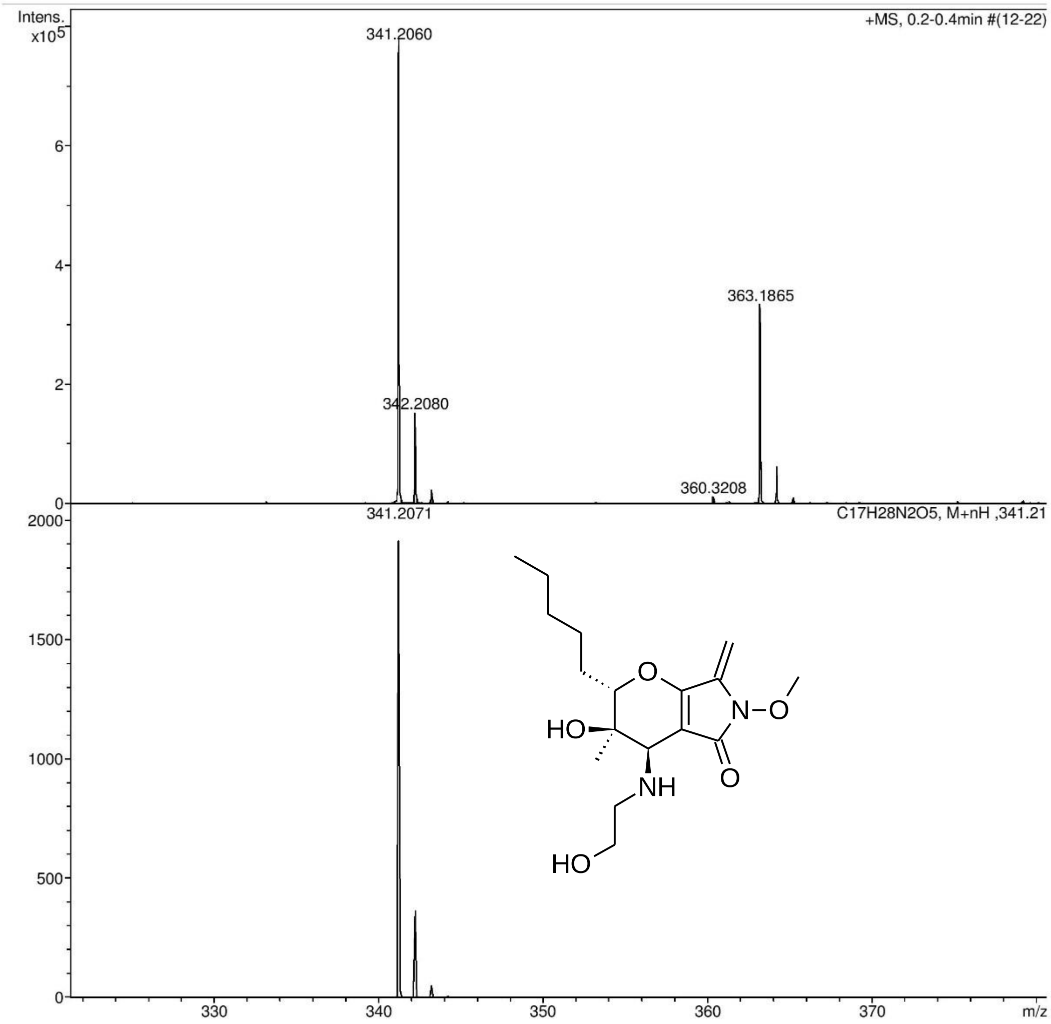

Bruker Compass DataAnalysis 4.0 printed: 08.02.2018 19:22:28 Page 1 of 1

Figure 8. HRMS of **2**.

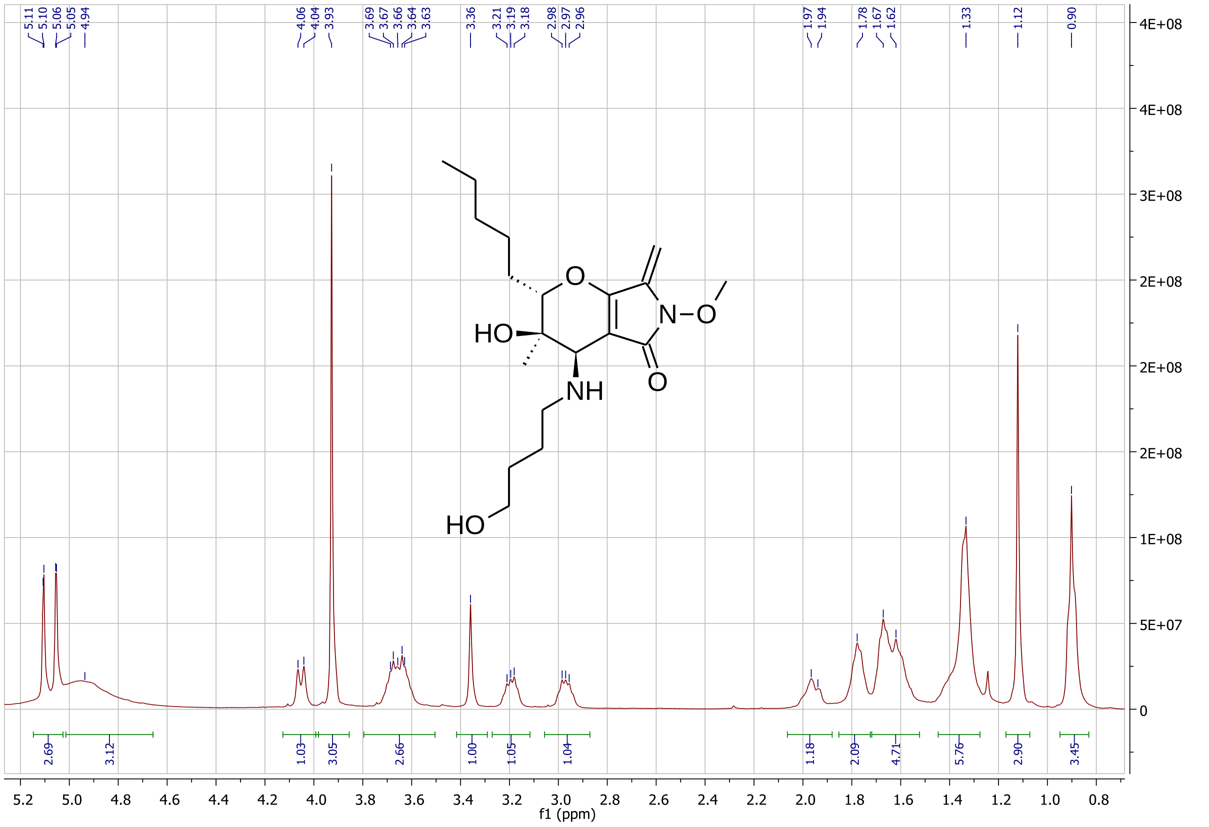

Figure 9. ^{1}H-NMR of **3**.

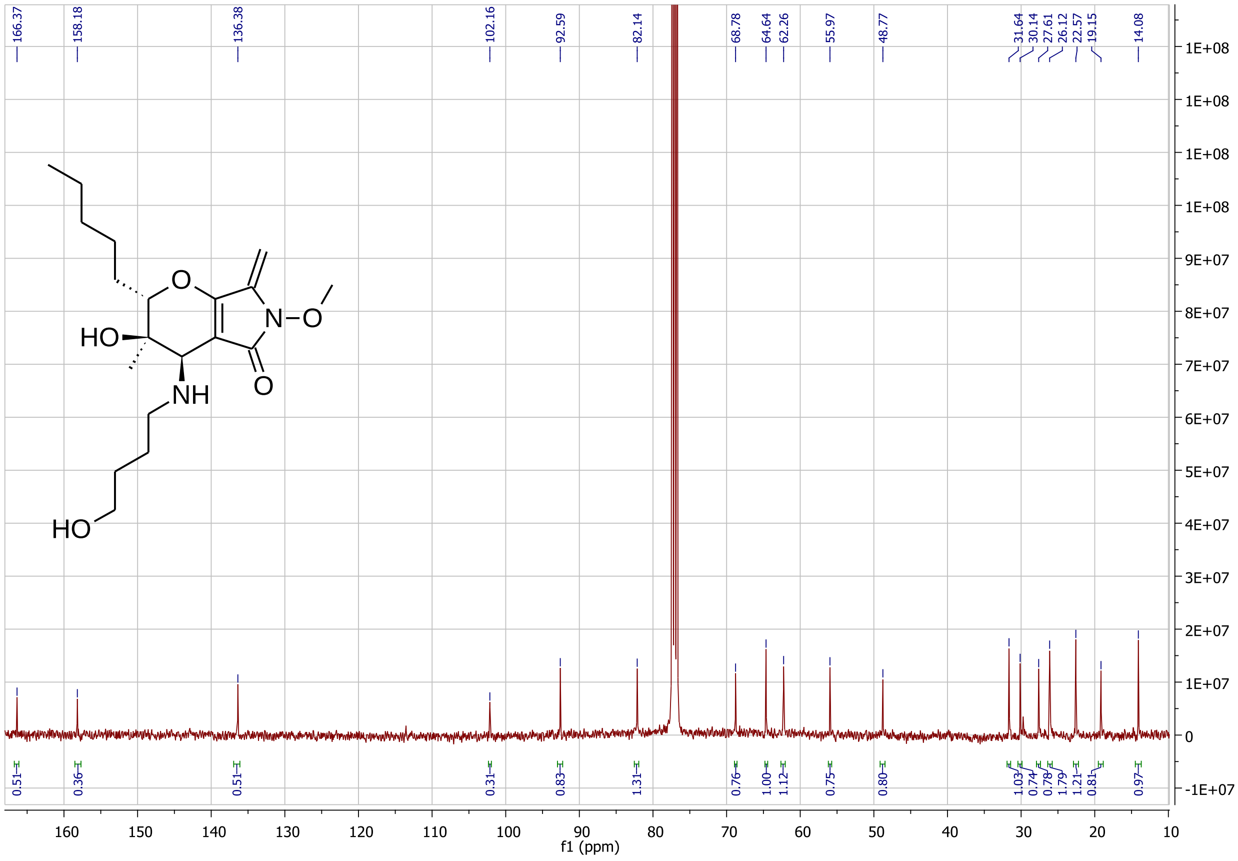

Figure 10. ^{13}C-NMR of **3**.

Display Report

Analysis Info

Analysis Name	D:\Data\2018\february\08\abz17.d	Acquisition Date	08.02.2018 19:28:11
Method	tune_low.m	Operator	Bruker Customer
Sample Name	abz17	Instrument / Ser#	micrOTOF 10223
Comment	MEOH		

Acquisition Parameter

Source Type	ESI	Ion Polarity	Positive	Set Nebulizer	0.4 Bar
Focus	Not active			Set Dry Heater	180 °C
Scan Begin	50 m/z	Set Capillary	4500 V	Set Dry Gas	4.0 l/min
Scan End	3000 m/z	Set End Plate Offset	-500 V	Set Divert Valve	Source

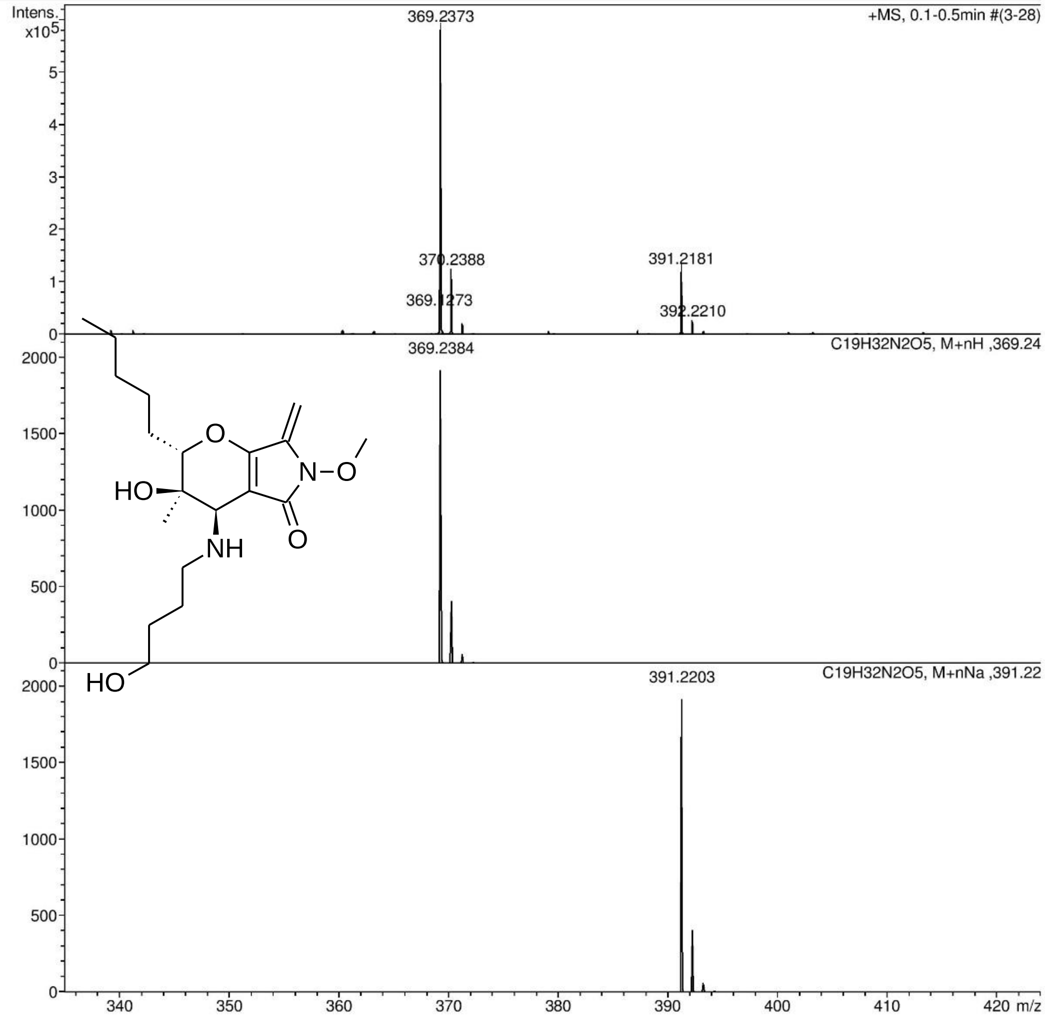

Bruker Compass DataAnalysis 4.0 printed: 08.02.2018 19:33:36 Page 1 of 1

Figure 11. HRMS of **3**.

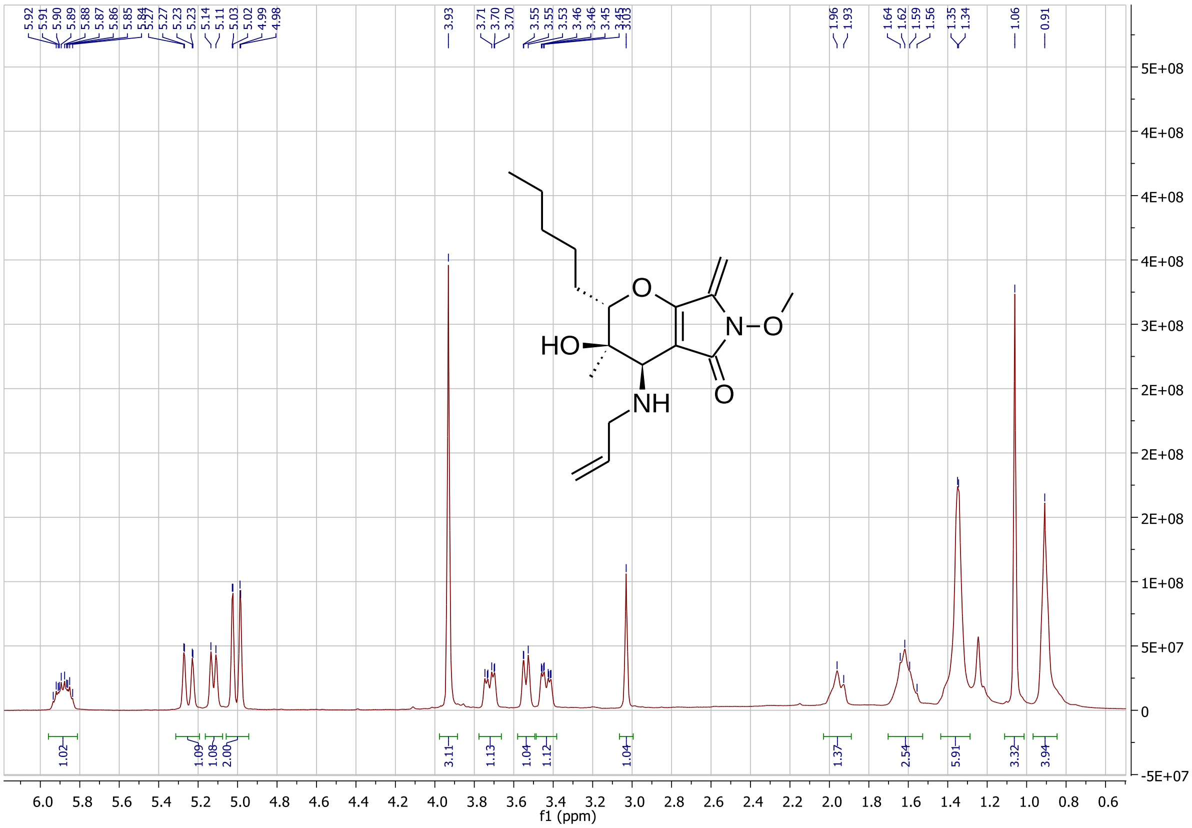

Figure 12. ^{1}H-NMR of **4**.

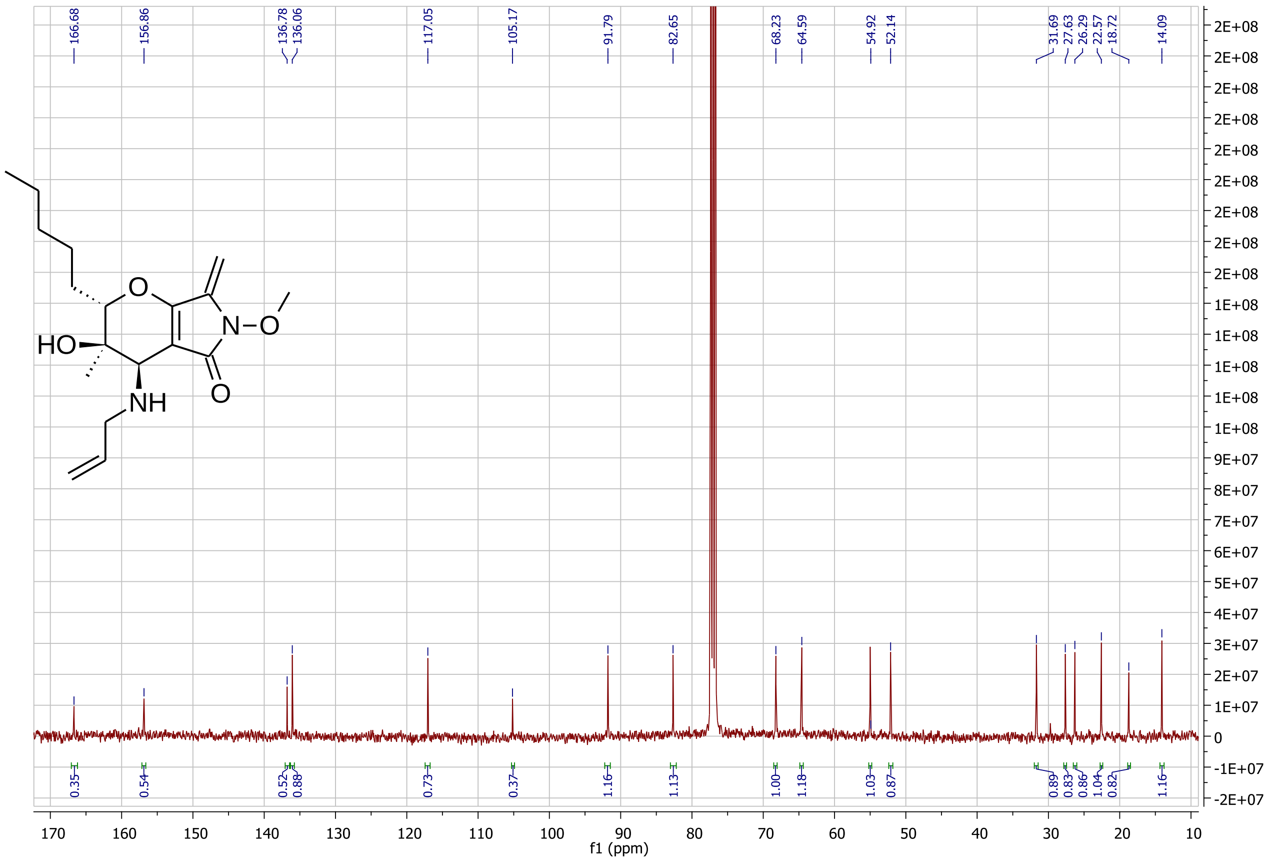

Figure 13. ^{13}C-NMR of **4**.

Display Report

Analysis Info

Analysis Name	D:\Data\2018\february\12\abz18.d	Acquisition Date	12.02.2018 15:27:58
Method	tune_low.m	Operator	Bruker Customer
Sample Name	abz18	Instrument / Ser#	micrOTOF 10223
Comment	MeOH		

Acquisition Parameter

Source Type	ESI	Ion Polarity	Positive	Set Nebulizer	0.4 Bar
Focus	Not active			Set Dry Heater	180 °C
Scan Begin	50 m/z	Set Capillary	4500 V	Set Dry Gas	4.0 l/min
Scan End	1500 m/z	Set End Plate Offset	-500 V	Set Divert Valve	Source

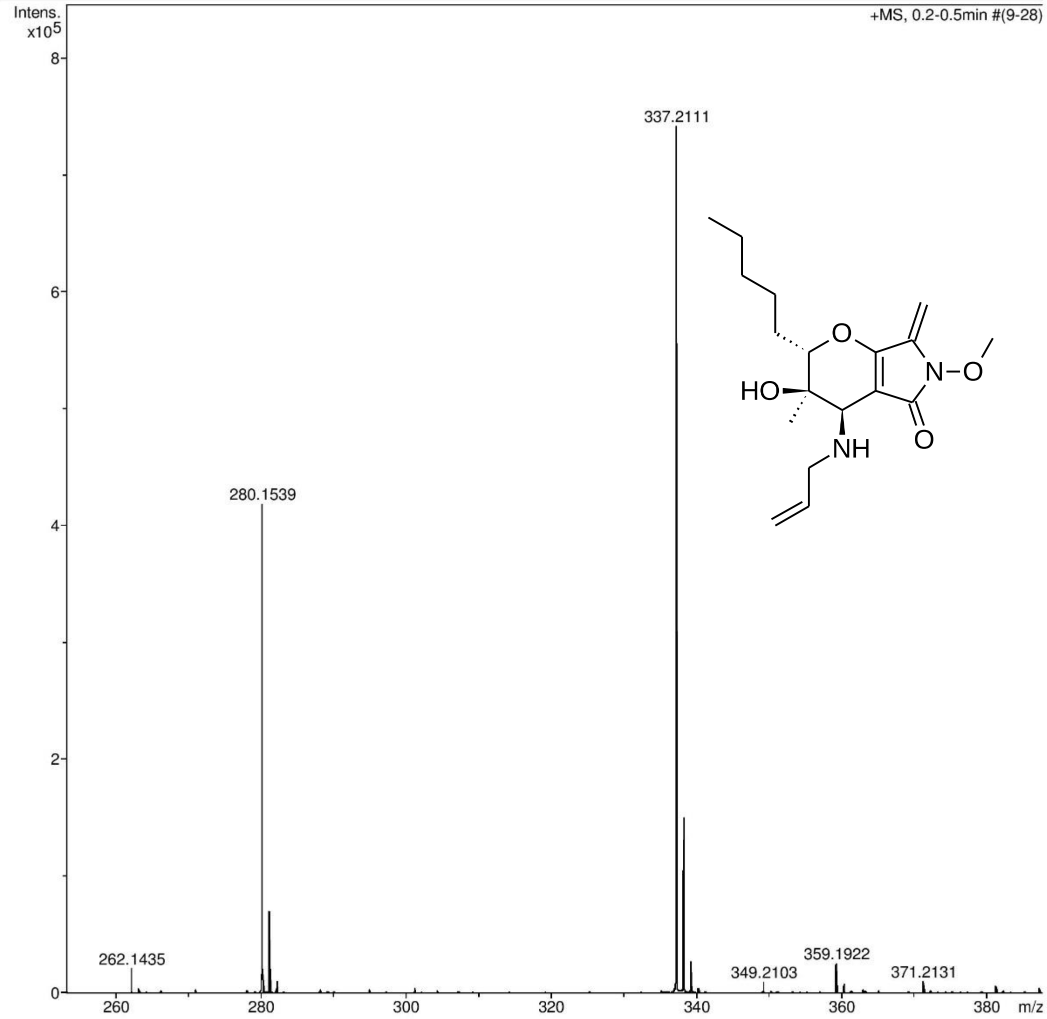

Bruker Compass DataAnalysis 4.0 printed: 12.02.2018 15:30:32 Page 1 of 1

Figure 14. HRMS of **4**.

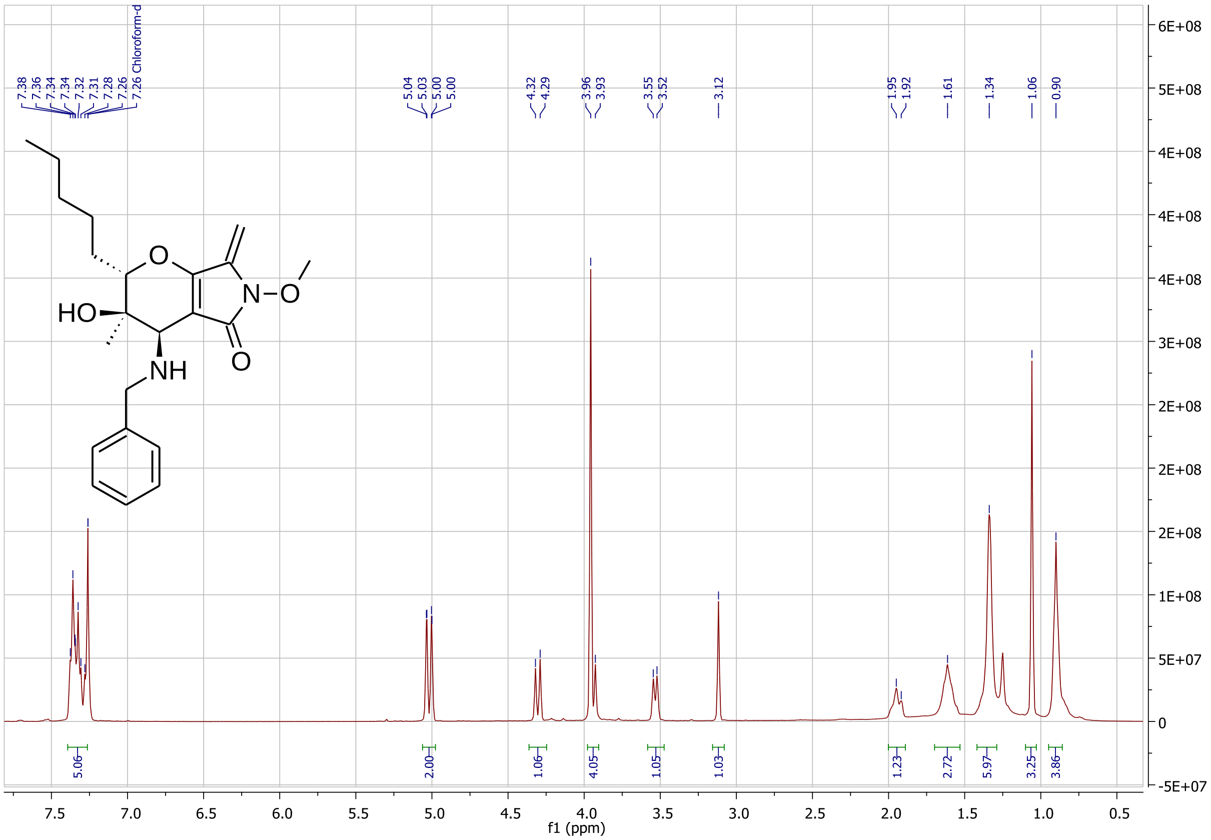

Figure 15. ^{1}H-NMR of **5**.

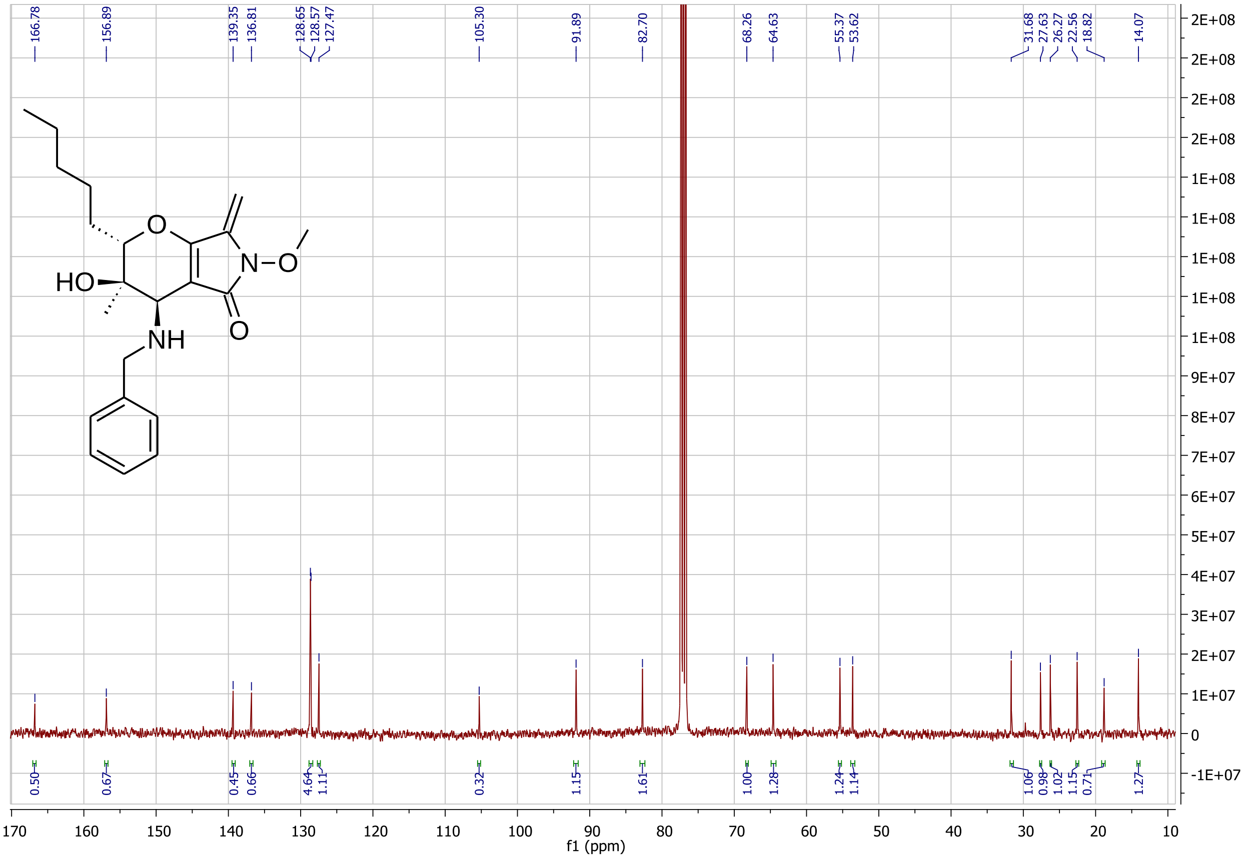

Figure 16. ^{13}C-NMR of **5**.

Display Report

Analysis Info

Analysis Name	D:\Data\2018\february\12\abz19.d	Acquisition Date	12.02.2018 15:35:22
Method	tune_low.m	Operator	Bruker Customer
Sample Name	abz19	Instrument / Ser#	micrOTOF 10223
Comment	MeOH		

Acquisition Parameter

Source Type	ESI	Ion Polarity	Positive	Set Nebulizer	0.4 Bar
Focus	Not active			Set Dry Heater	180 °C
Scan Begin	50 m/z	Set Capillary	4500 V	Set Dry Gas	4.0 l/min
Scan End	1500 m/z	Set End Plate Offset	-500 V	Set Divert Valve	Source

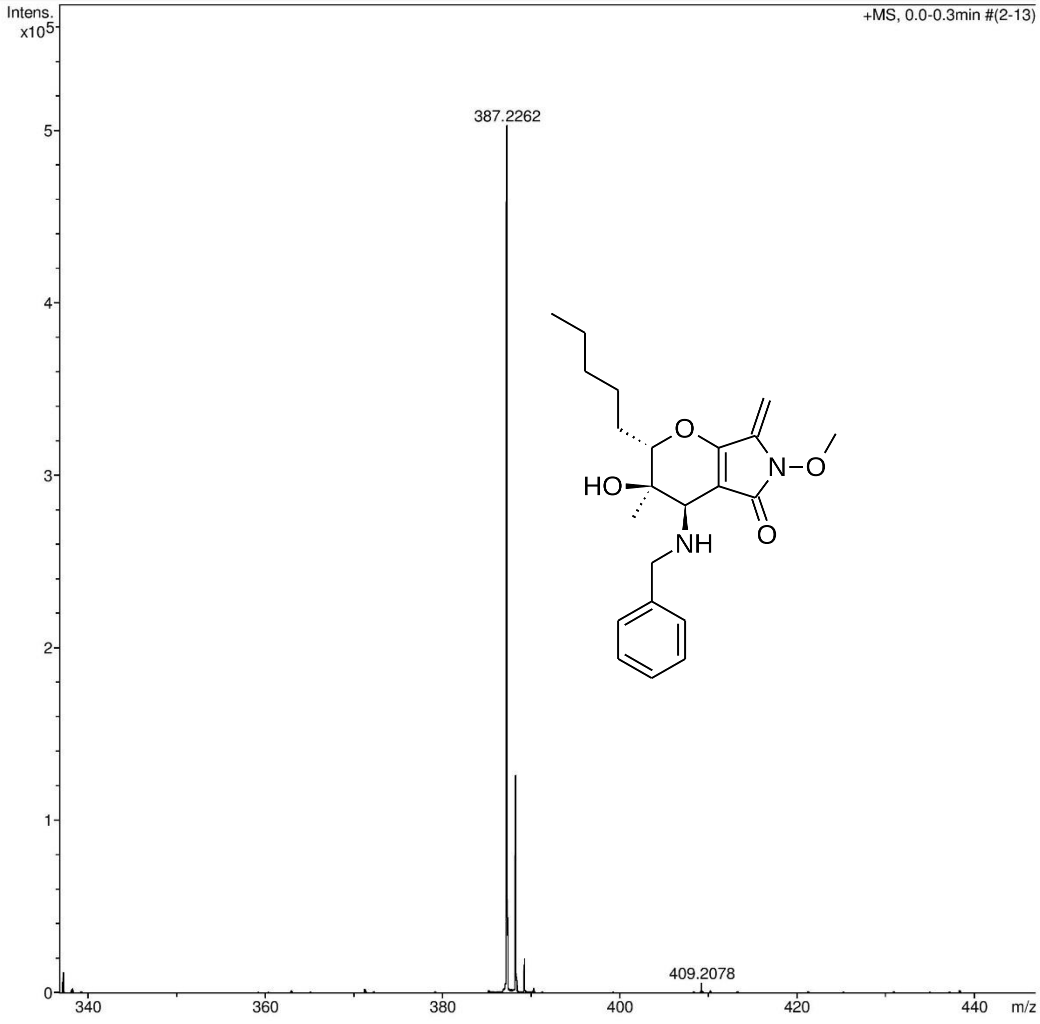

Bruker Compass DataAnalysis 4.0 printed: 12.02.2018 15:37:53 Page 1 of 1

Figure 17. HRMS of **5**.

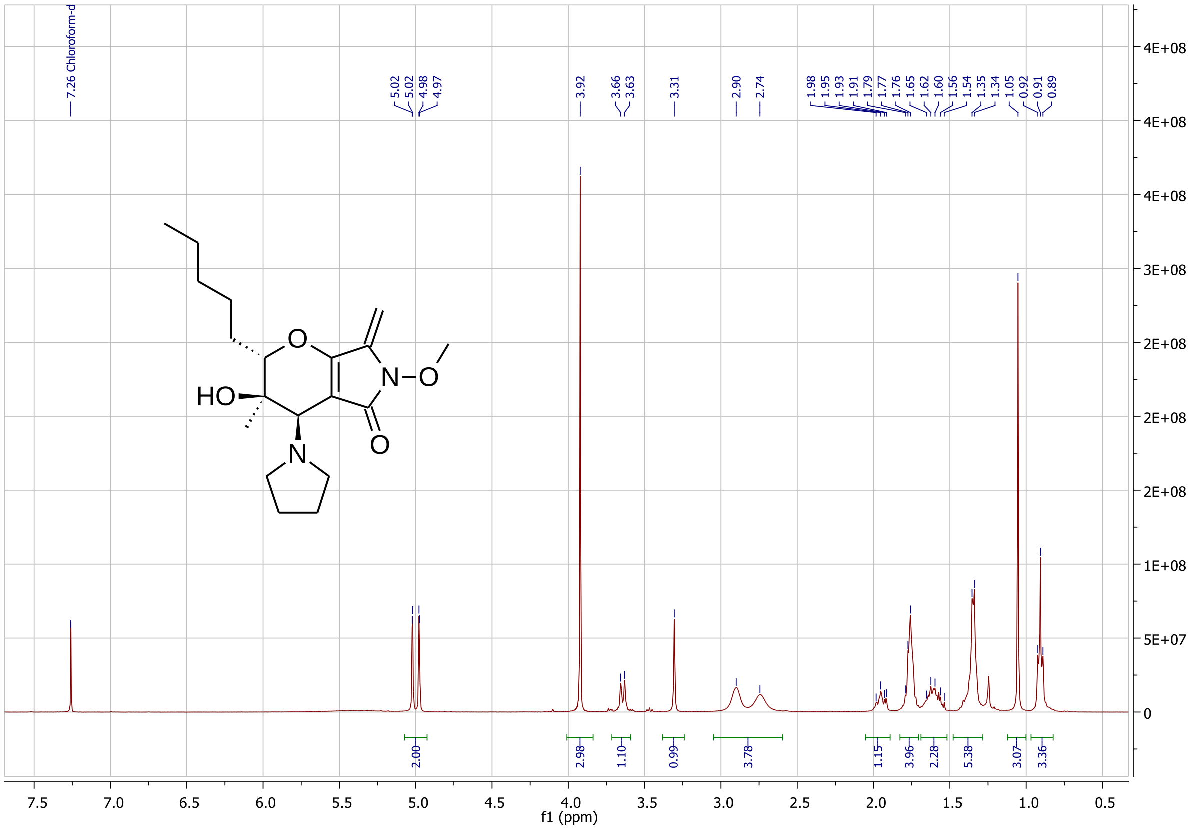

Figure 18. ^{1}H-NMR of **6**.

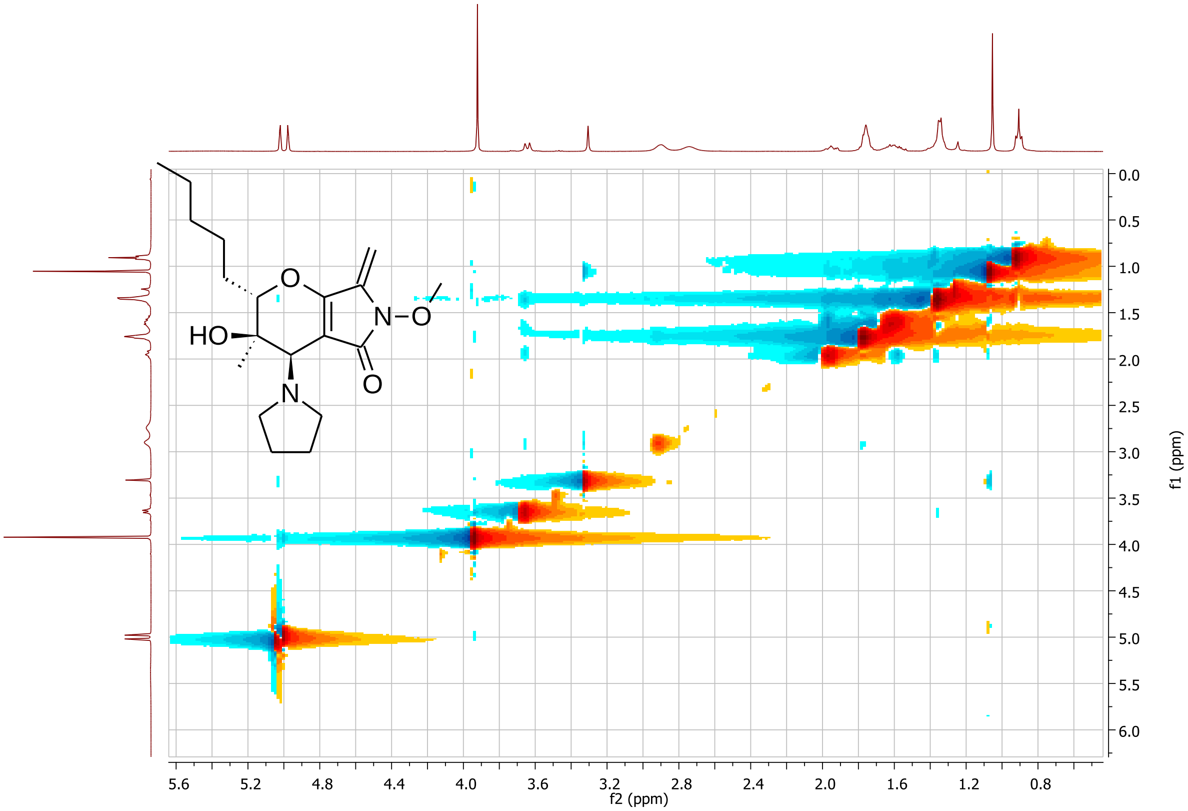

Figure 19. ^{1}H-^{1}H ROESY of **6**.

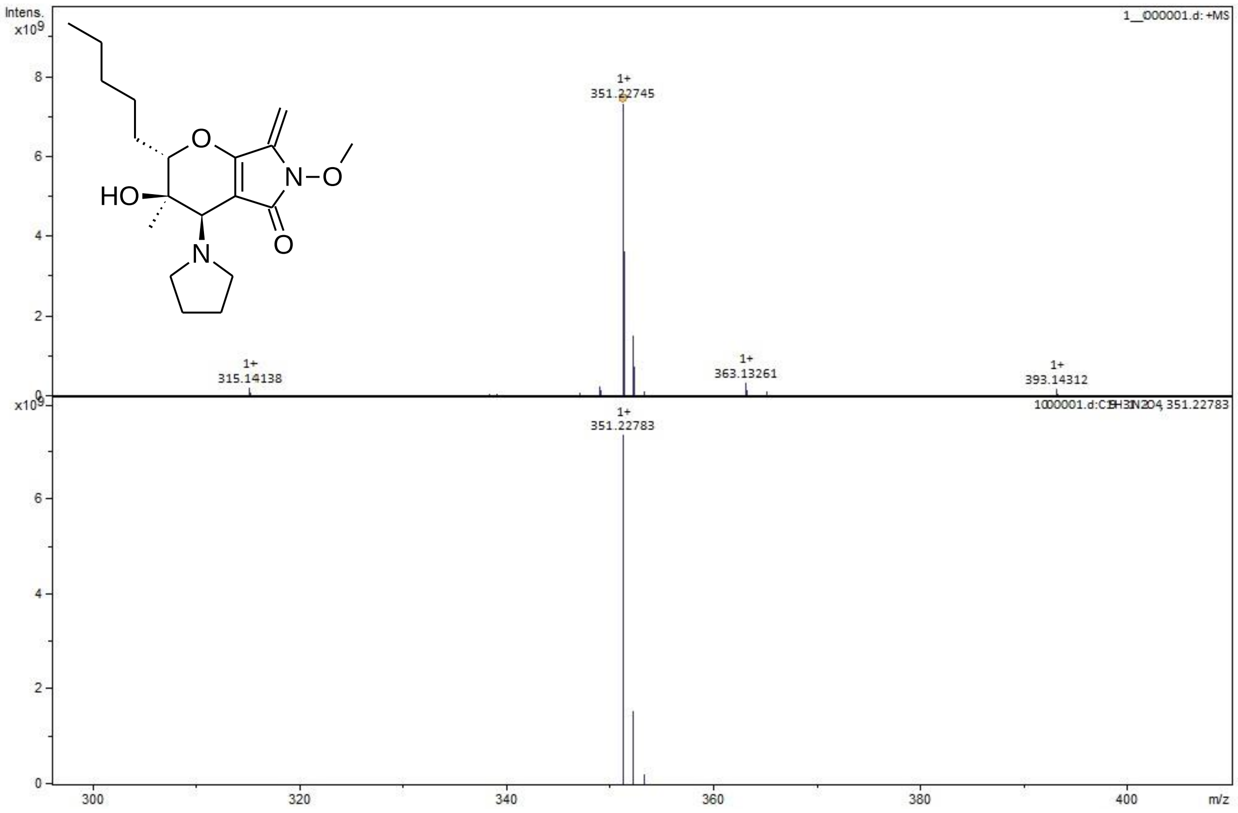

Figure 20. HRMS of **6**.

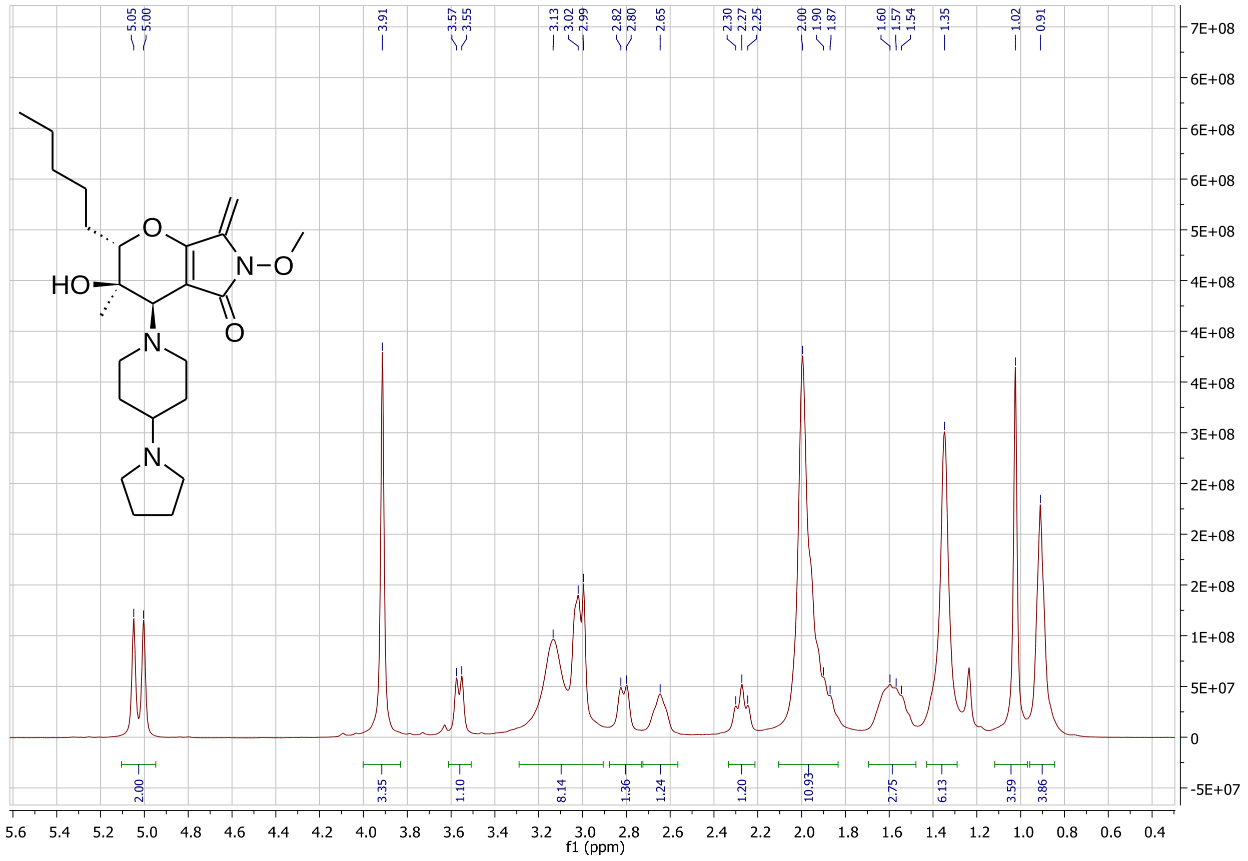

Figure 21. ^{1}H-NMR of **7**.

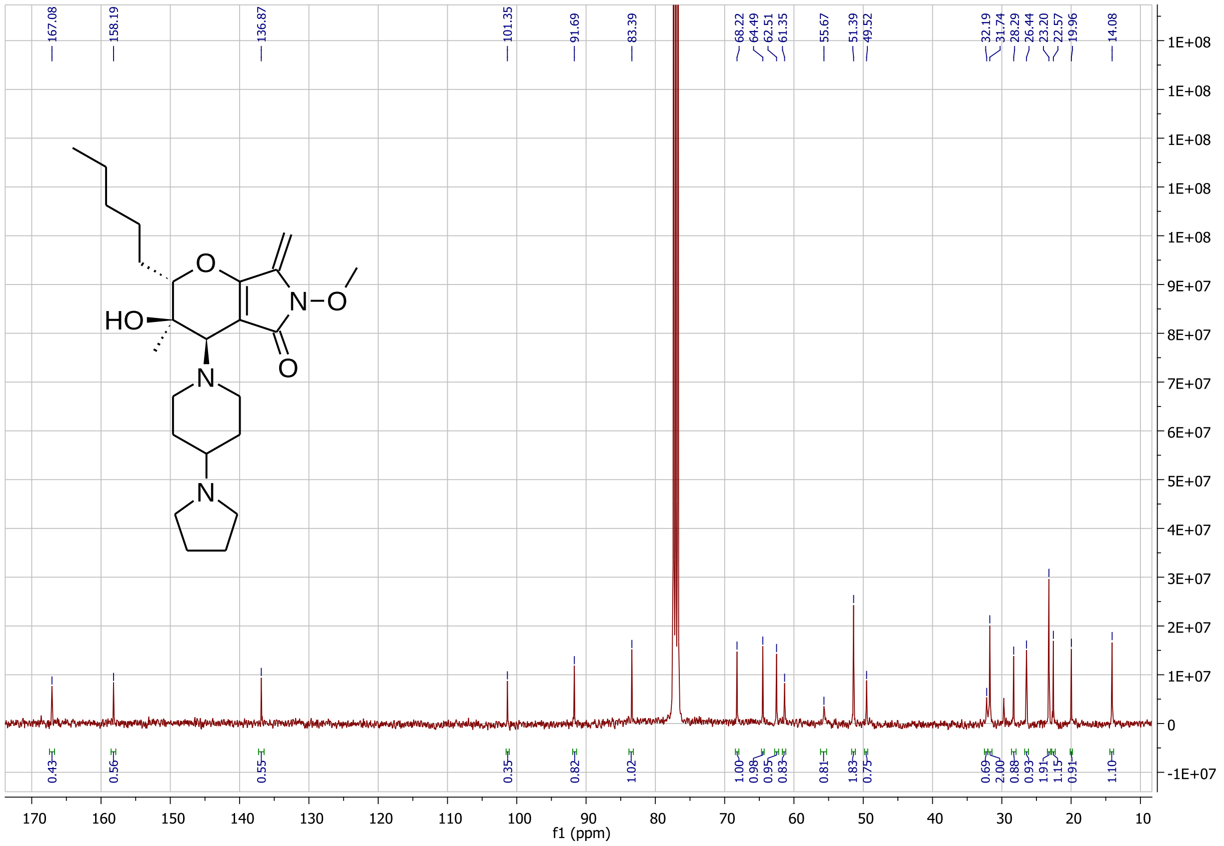

Figure 22. ^{13}C-NMR of **7**.

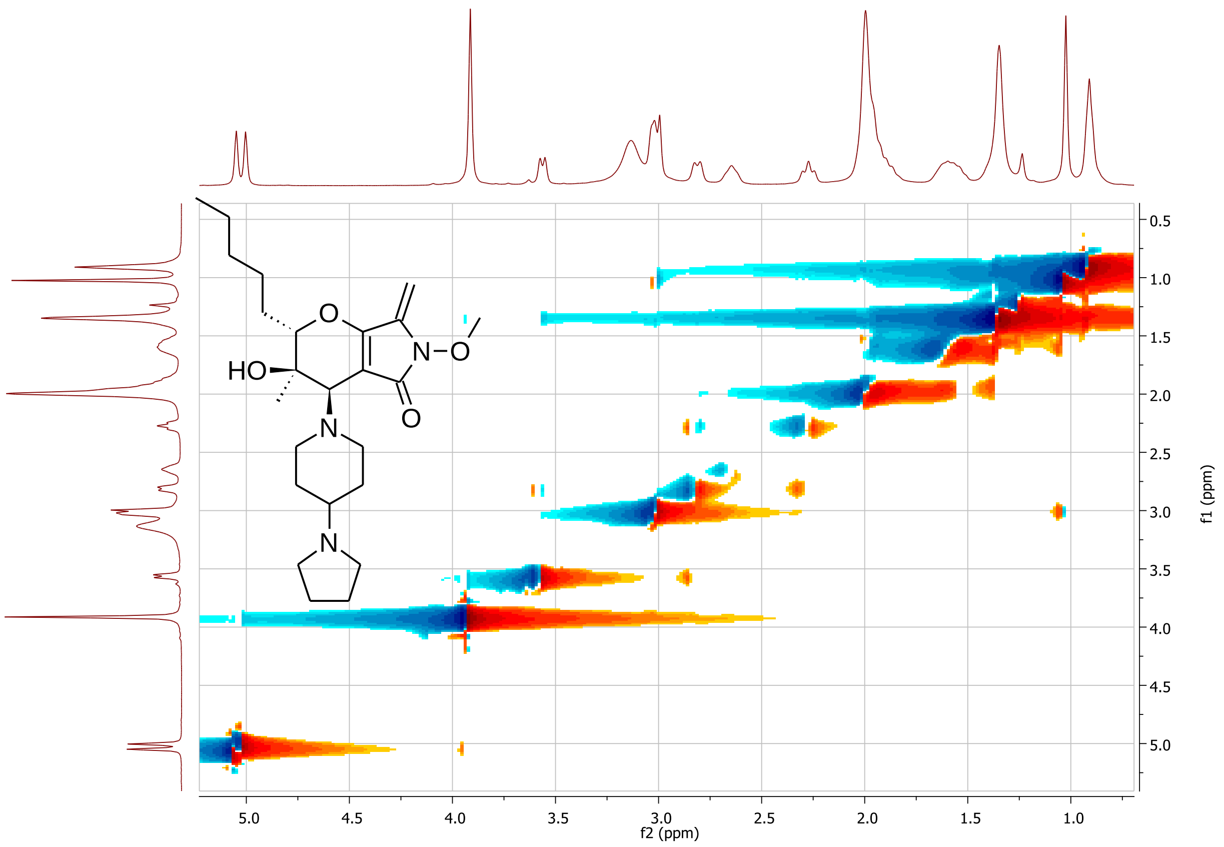

Figure 23. ^{1}H-^{1}H ROESY of **7**.

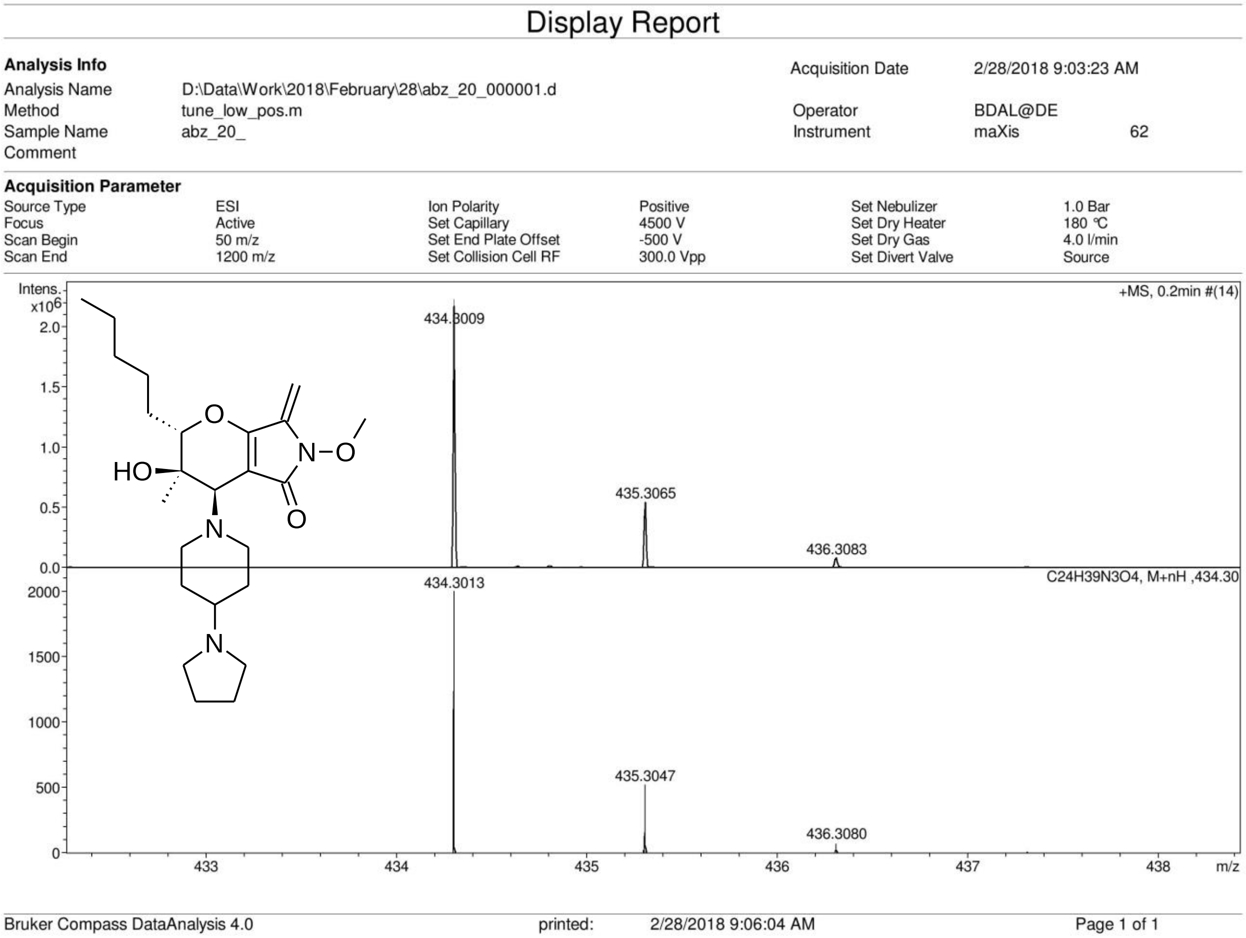

Figure 24. HRMS of **7**.

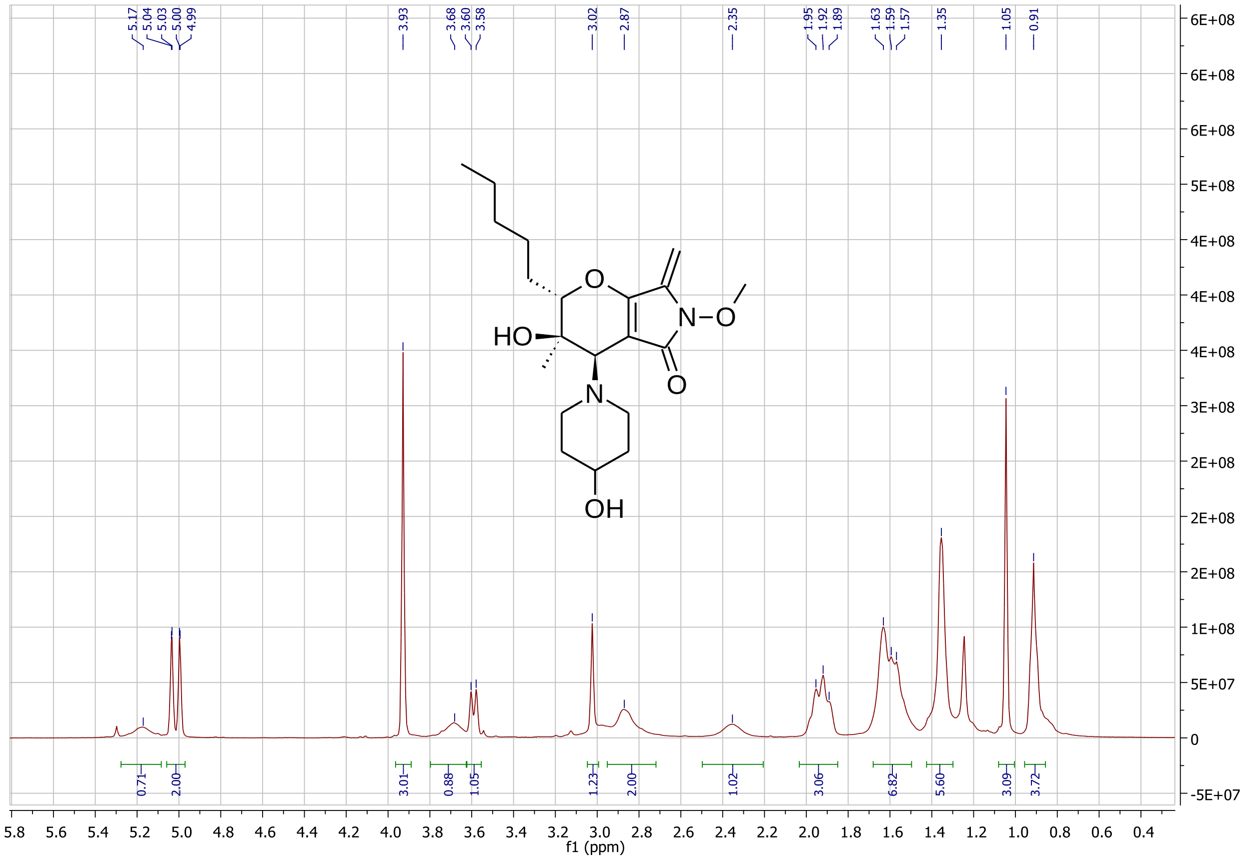

Figure 25. ^{1}H-NMR of **8**.

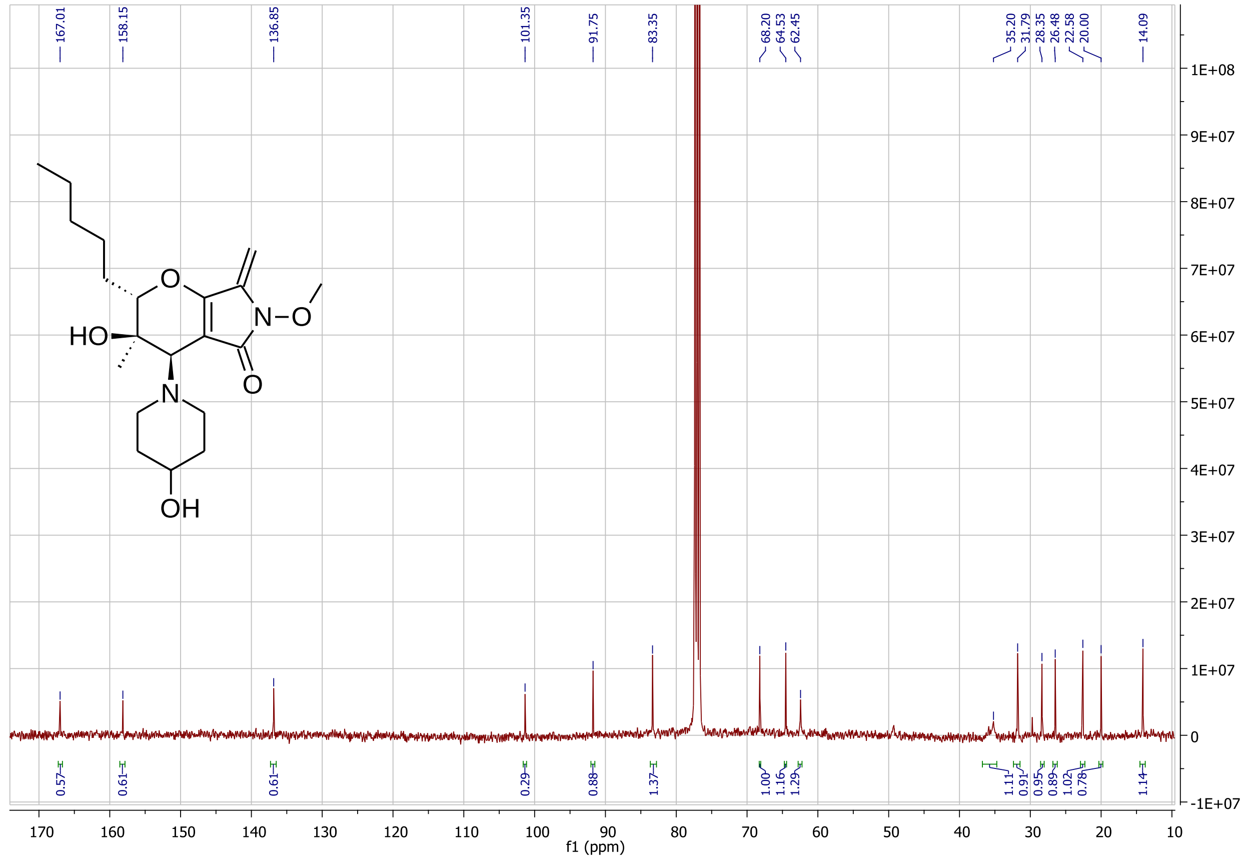

Figure 26. ^{13}C-NMR of **8**.

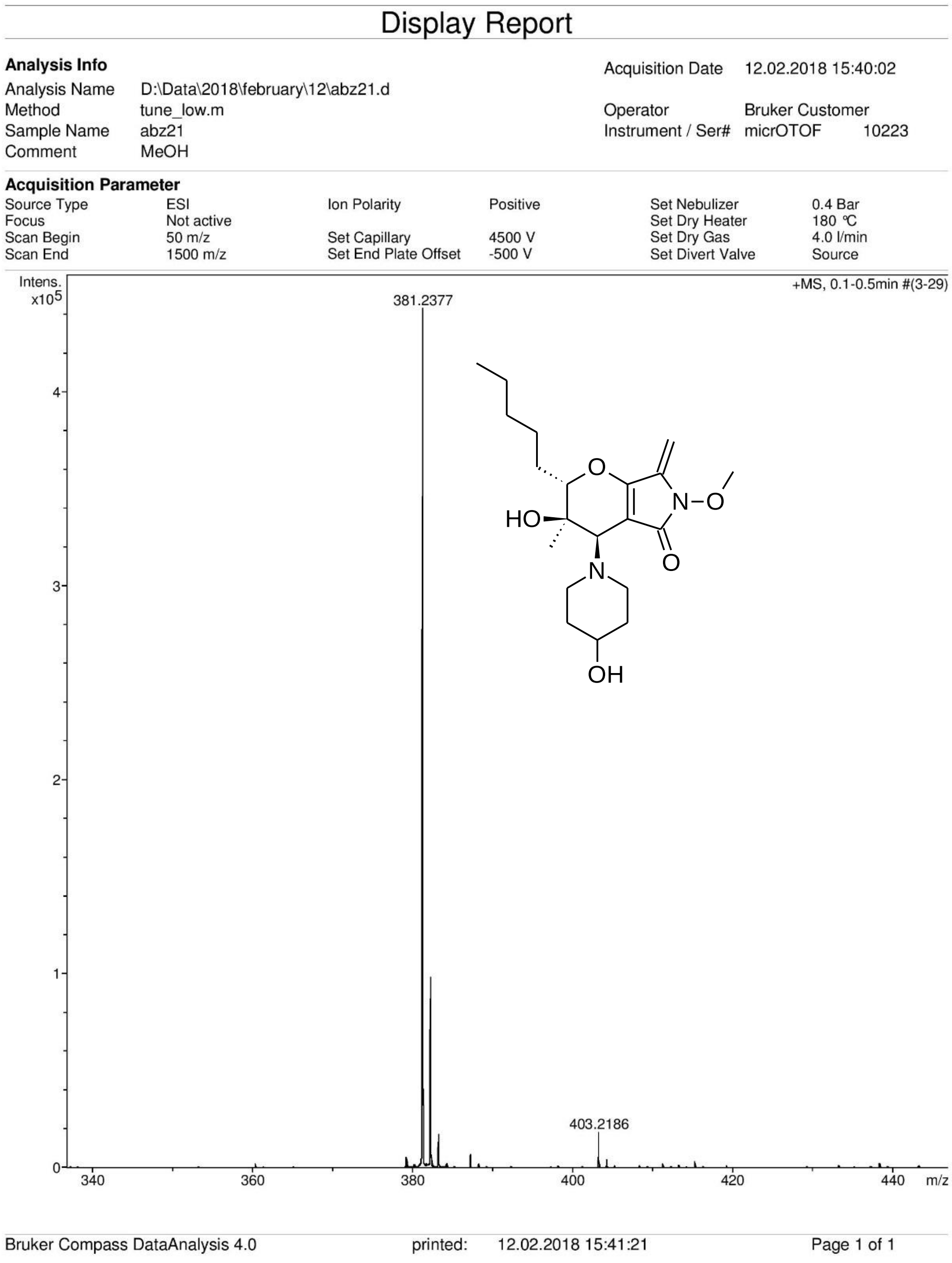

Figure 27. HRMS of **8**.
